# Associations of Mediterranean Diet Adherence with Cardiometabolic Outcomes, Psychological Symptoms, and Health-Related Quality of Life Among Adults Attending Primary Care Centres in Navarra, Spain: A Cross-Sectional Study

**DOI:** 10.3390/nu18193156

**Published:** 2026-09-25

**Authors:** Julieth Paola Sulaiman Rojas, José Abellán Alemán, Gonzalo Luis Alonso Salinas, Miladi del Socorro Golac Rabanal, Cédric Marco Detchart, Isabel Arrechea Irigoyen, Manuela García Sánchez, Daime Pérez Feíto, Virginia Íñiguez García, Roberto Dair García de la Rosa, Grethel Zarimah Díaz Vargas, Hector Orellana-Arauco, María Lourdes Azcona Cortés, Yohanna Elizabeth Barría Jujihara, Julienne Ntumba Mumba, Martín Andrés Astudillo Astudillo, Carlos José Zhuzhingo Vásquez, Javier Nieto Iglesias, Luis Alberto Díaz Vera, Alcibíades Segundo Díaz Vera

**Affiliations:** 1 International Doctoral School, Catholic University of Murcia (UCAM), 30107 Murcia, Spainmaa3studillo@hotmail.com (M.A.A.A.); 2 Institute for Health Research of Navarra (IdiSNA), 31008 Pamplona, Spainrdairg@gmail.com (R.D.G.d.l.R.);; 3 Department of Health Sciences, Public University of Navarra (UPNA), 31006 Pamplona, Spain; lur.azcona@gmail.com (M.L.A.C.); yohanna.jujihara@gmail.com (Y.E.B.J.);; 4 Faculty of Health Sciences, Catholic University of Murcia (UCAM), 30107 Murcia, Spain; 5 Department of Cardiology, Hospital Universitario de Navarra, 31008 Pamplona, Spain; 6 Navarrabiomed, Fundación Miguel Servet, 31008 Pamplona, Spain; 7 Primary Care, Navarra Health Service–Osasunbidea, 31003 Pamplona, Spain; 8 Department of Statistics, Computer Science and Mathematics, Public University of Navarra (UPNA), 31006 Pamplona, Spain; 9 Faculty of Medicine, Universidad Peruana Cayetano Heredia, Lima 15102, Peru; diazvera19@gmail.com; 10 Department of Cardiology and Coronary Care, Hospital Nacional Arzobispo Loayza, Lima 15082, Peru; 11 Academic Department, Faculty of Dentistry, Universidad Nacional Federico Villarreal, Lima 15088, Peru; 12 Hospital Universitario San Pedro, 26006 Logroño, Spain; 13 Hospital General Universitario de Ciudad Real, Servicio de Salud de Castilla-La Mancha (SESCAM), 13005 Ciudad Real, Spain; 14 Hospital Nacional Edgardo Rebagliati Martins, Lima 15072, Peru; 15 Faculty of Health Sciences, Universidad Internacional de Valencia (VIU), C/ Pintor Sorolla, 21, 46002 Valencia, Spain

**Keywords:** Mediterranean diet, MEDAS-14, blood pressure, fasting blood glucose, anxiety, health-related quality of life, primary care, cross-sectional study

## Abstract

**Background/Objectives:** Mediterranean diet adherence has been associated with cardiometabolic and psychological health, but evidence integrating these outcomes with health-related quality of life in primary care populations remains limited. This study examined the associations between Mediterranean diet adherence and anthropometric, cardiometabolic, psychological, and health-related quality-of-life outcomes. **Methods:** This cross-sectional study included 495 adults attending primary care centres in Navarra, Spain. Mediterranean diet adherence was assessed using the validated 14-item Mediterranean Diet Adherence Screener (MEDAS-14). Multivariable linear regression models examined associations between the continuous MEDAS-14 score and study outcomes, adjusting for age, sex, educational attainment, physical activity, and BMI when appropriate. Sensitivity analyses, assessment of non-linearity, false-discovery-rate correction across the nine principal outcomes, and interaction-by-sex analyses were also performed. **Results:** Each one-point increase in the MEDAS-14 score was associated with lower systolic blood pressure (β = −1.056 mmHg; 95% confidence interval (CI): −1.774 to −0.338; *p* = 0.004), diastolic blood pressure (β = −0.838 mmHg; 95% CI: −1.261 to −0.415; *p* < 0.001), fasting blood glucose (β = −0.745 mg/dL; 95% CI: −1.365 to −0.125; *p* = 0.019), and Goldberg anxiety score (β = −0.249; 95% CI: −0.368 to −0.130; *p* < 0.001), and with higher EQ VAS scores (β = 1.224; 95% CI: 0.570 to 1.878; *p* < 0.001). The associations with BMI, systolic and diastolic blood pressure, fasting blood glucose, Goldberg anxiety score, and EQ VAS remained statistically evident after false-discovery-rate correction, and the overall pattern was generally consistent across sensitivity analyses. No adjusted association with Goldberg depression score was observed. BMI was inversely associated with MEDAS-14 in the principal linear model (β = −0.299 kg/m^2^; 95% CI: −0.521 to −0.077; *p* = 0.009), whereas the association with waist circumference was not statistically significant (β = −0.475 cm; 95% CI: −1.046 to 0.095; *p* = 0.102). BMI, waist circumference, and total cholesterol showed evidence of non-linearity. Exploratory interaction analyses showed evidence of interaction by sex for diastolic blood pressure and EQ VAS after false-discovery-rate correction. **Conclusions:** Higher MEDAS-14 scores were associated with lower blood pressure and fasting blood glucose, fewer anxiety symptoms, and better self-perceived health. Associations for BMI, waist circumference, and total cholesterol showed evidence of non-linearity and should be interpreted cautiously. These cross-sectional findings do not establish temporality or causality and should also be interpreted in light of the lack of adjustment for relevant clinical diagnoses and medication use. Prospective confirmation is warranted.

## 1. Introduction

The traditional Mediterranean diet is characterised by abundant consumption of vegetables, fruits, legumes, nuts, cereals, and olive oil; low-to-moderate consumption of fish, poultry, and dairy products; and relatively low consumption of red meat and saturated fat [1]. Rather than representing an isolated collection of foods, it constitutes an overall dietary and lifestyle pattern in which food combinations, culinary practices, physical activity, and social behaviours may jointly contribute to its health-related characteristics [1]. Greater adherence to this dietary pattern has been associated with lower all-cause mortality and a reduced risk of cardiovascular, metabolic, and other chronic diseases [2,3,4,5].

The risk of cardiovascular disease is strongly influenced by modifiable factors, including excess adiposity, elevated blood pressure, impaired glucose regulation, dyslipidaemia, smoking, physical inactivity, and poor diet quality [3,4,5]. The Mediterranean dietary pattern provides fibre, unsaturated fatty acids, polyphenols, vitamins, minerals, and other bioactive compounds that may favourably influence body weight, endothelial function, inflammation, oxidative stress, insulin sensitivity, and blood pressure regulation [3]. Nevertheless, associations with individual cardiometabolic outcomes are not always consistent, particularly for total cholesterol and anthropometric measurements, and may vary according to population characteristics, adherence levels, dietary assessment methods, and analytical approaches [3,4,5].

Beyond cardiometabolic health, Mediterranean diet adherence may also be associated with psychological well-being and health-related quality of life. Previous research has linked greater adherence to fewer anxiety and depressive symptoms, although the consistency and strength of the evidence vary according to study design, population, dietary assessment method, and psychological instrument [6]. Greater adherence has also generally been associated with better health-related quality of life in adult populations [6,7]. However, evidence simultaneously evaluating cardiometabolic, psychological, and health-related quality-of-life outcomes within the same primary care population remains limited.

Primary care represents an important setting for identifying dietary behaviours associated with potentially modifiable health outcomes. Brief dietary screening instruments may be particularly useful in this context because they can be incorporated into routine consultations without the burden of a comprehensive dietary assessment [8]. The 14-item Mediterranean Diet Adherence Screener (MEDAS-14), developed within the PREDIMED study and validated against a comprehensive food-frequency questionnaire, provides a rapid estimate of adherence to the Mediterranean dietary pattern [8,9]. The instrument comprises 14 binary components, each scored as 0 or 1, producing a total score ranging from 0 to 14, with higher scores indicating greater adherence [8,9].

Mediterranean diet adherence may also be socially and behaviourally patterned. Age, sex, educational attainment, physical activity, smoking, and alcohol consumption may be related to both dietary adherence and health outcomes. Sex may additionally modify the associations between diet and cardiometabolic or well-being outcomes. Furthermore, assuming a constant linear association across the complete range of the adherence score may obscure more complex relationships, particularly when relatively few participants have scores at the extremes of the distribution.

The primary objective of the MEDINAV study was to examine the adjusted associations between the continuous MEDAS-14 score and anthropometric, cardiometabolic, psychological, and health-related quality-of-life outcomes among adults attending primary care centres in Navarra, Spain. The outcomes examined were body mass index, waist circumference, systolic and diastolic blood pressure, fasting blood glucose, total cholesterol, Goldberg anxiety and depression scores, and EuroQol visual analogue scale (EQ VAS) score.

The secondary objectives were to describe the distribution of the 14 individual MEDAS-14 components; evaluate differences in adherence according to sociodemographic, lifestyle, and anthropometric characteristics; compare study outcomes across low-, moderate-, and high-adherence categories; assess the robustness and potential non-linearity of the associations; and explore effect modification by sex.

We hypothesised that higher MEDAS-14 scores would be associated with lower adiposity, more favourable blood pressure and glucose profiles, fewer anxiety and depressive symptoms, and better self-perceived health. Given the cross-sectional design, the study was designed to estimate associations rather than causal effects.

## 2. Materials and Methods

### 2.1. Study Design and Setting

The Mediterranean Diet Study in the Adult Population of Navarra (MEDINAV) was an observational, cross-sectional study conducted in primary care centres of the Navarra Health Service–Osasunbidea in Navarra, Spain. Participant recruitment and data collection took place from June 2024 to December 2025. The study is reported in accordance with the Strengthening the Reporting of Observational Studies in Epidemiology (STROBE) statement and its extension for nutritional epidemiology (STROBE-nut) [10,11].

### 2.2. Participants

Participants were recruited from five primary care centres in Navarra, Spain. Adults aged ≥18 years attending routine primary care consultations were consecutively approached and assessed for eligibility. During the recruitment period, all patients attending the participating centres who met the eligibility criteria were invited to participate. Recruitment was not quota-based according to centre, sex, age group, or consultation type.

Eligibility criteria included residence in Navarra for at least one year, the ability and willingness to provide written informed consent, willingness to complete the study assessments, and not following a prescribed or structured therapeutic diet for health-related reasons. This criterion did not exclude individuals who had previously received general dietary advice for conditions such as hypertension, diabetes, dyslipidaemia, or obesity.

Exclusion criteria were an acute condition or severe chronic disease requiring a specific diet or potentially affecting usual dietary habits; a major dietary change during the preceding six months; pregnancy or breastfeeding; a diagnosed eating disorder; or any cognitive, linguistic, or health-related limitation preventing comprehension or completion of the study assessments.

A total of 504 adults were approached and assessed for participation. Two individuals were excluded because their cancer diagnoses met the criterion of severe chronic disease potentially affecting usual dietary habits, and seven individuals declined to participate. The final analytical sample comprised 495 participants (Figure 1).

An a priori sample-size calculation was performed for a fixed-model multiple linear regression analysis based on the increase in R^2^, with one tested predictor and up to eight total predictors. Assuming a small incremental effect size of *f*^2^ = 0.02, 80% statistical power, and a two-sided α level of 0.05, the minimum required sample was estimated at 395 participants. The final analytical sample of 495 participants exceeded this requirement.

### 2.3. Data Collection and Measurements

#### 2.3.1. Mediterranean Diet Adherence Score

Adherence to the Mediterranean diet was assessed using the validated 14-item Mediterranean Diet Adherence Screener (MEDAS-14), developed within the PREDIMED study [8,9]. The instrument comprises 12 questions on food consumption frequency and two questions on dietary habits. Each component was scored as 0 or 1 according to the validated criteria, producing a total score ranging from 0 to 14, with higher scores indicating greater adherence. The complete scoring criteria for all 14 components are presented in Supplementary Table S1.

The MEDAS-14 score was analysed primarily as a continuous exposure variable. For descriptive purposes and secondary categorical analyses, participants were classified as having low (≤7 points), moderate (8–9 points), or high (≥10 points) adherence, consistent with categories previously used in PREDIMED-related analyses [9]. Because these cut-offs do not represent independently validated clinical thresholds, all categorical analyses were considered exploratory.

#### 2.3.2. Sociodemographic and Lifestyle Variables

Sociodemographic variables included age, sex, and educational attainment. Age was analysed as a continuous variable and was also grouped into 18–39, 40–59, and ≥60 years for descriptive analyses. Educational attainment was classified as basic education, secondary or vocational education, or university education or higher.

Physical activity was assessed using the seven-item short form of the International Physical Activity Questionnaire (IPAQ), referring to activity performed during the previous seven days [12]. Participants were classified as having low/inactive, moderate, or high physical activity according to the IPAQ scoring protocol.

Cigarette consumption was classified according to the reported number of cigarettes smoked per day as none, <10, 11–20, 21–30, or >30 cigarettes/day. For the sensitivity analyses, current smoking was defined as any reported current cigarette consumption, with participants reporting no current consumption classified as non-current smokers.

Alcohol-consumption frequency was classified as never, once a month or less, two to four times per month, two to four times per week, or four or more times per week. The number of alcoholic drinks consumed per drinking day was retained in its original questionnaire categories: 1–2, 3–4, 5–6, 7–8, or ≥10 drinks. Participants who reported never consuming alcohol were coded as 0 (not applicable) for this variable. Because these response categories represented ranges rather than exact quantities, the number of drinks consumed per drinking day was described categorically and was not analysed as a quantitative measure of total alcohol intake. For sensitivity analyses, alcohol consumption was additionally dichotomised as any versus no consumption based on the alcohol-consumption-frequency variable. The distributions and questionnaire response categories of the lifestyle variables are presented in Supplementary Table S6.

#### 2.3.3. Anthropometric and Clinical Outcomes

Anthropometric variables included body weight, height, body mass index (BMI), waist circumference, and hip circumference. BMI was calculated as weight in kilograms divided by height in metres squared and expressed in kg/m^2^. Waist and hip circumferences were expressed in centimetres. For descriptive analyses, BMI was classified according to World Health Organization criteria as underweight (<18.5 kg/m^2^), normal weight (18.5–24.9 kg/m^2^), overweight (25.0–29.9 kg/m^2^), or obesity (≥30.0 kg/m^2^) [13].

Clinical outcomes included systolic and diastolic blood pressure, expressed in mmHg; fasting blood glucose, expressed in mg/dL; and total cholesterol, expressed in mg/dL. At study inclusion, blood pressure was measured in all participants using a validated blood-pressure monitor. Before measurement, participants remained seated and rested for five minutes, with their backs supported and legs uncrossed. The cuff was placed on the bare upper arm at heart level, and participants were instructed not to speak during the procedure. Three consecutive blood-pressure readings were obtained. The first reading was discarded, and the mean of the second and third readings was used in the analyses.

All participants were instructed to undergo a 12 h overnight fast before blood sampling. A fasting blood test was performed at study inclusion. All blood samples were processed within the standardized laboratory system of the Navarra Health Service, and fasting blood glucose and total cholesterol concentrations were determined using routine clinical laboratory procedures. The resulting clinical and laboratory values were recorded in the participants’ electronic health records and subsequently extracted for the present analyses.

#### 2.3.4. Psychological Outcomes

Anxiety and depressive symptoms were assessed using the Goldberg Anxiety and Depression Scale [14]. The instrument comprises separate nine-item anxiety and nine-item depression subscales. Each affirmative response was assigned one point, producing a score ranging from 0 to 9 for each subscale, with higher scores indicating a greater number of symptoms. The anxiety and depression subscale scores were analysed separately as continuous outcomes. The scale was used to assess psychological symptoms and was not intended to establish clinical psychiatric diagnoses.

#### 2.3.5. Health-Related Quality of Life

Health-related quality of life was evaluated using the EuroQol five-dimension, three-level questionnaire (EQ-5D-3L) and the EuroQol visual analogue scale (EQ VAS) [15]. The EQ-5D-3L evaluates mobility, self-care, usual activities, pain/discomfort, and anxiety/depression, with three response levels for each dimension. The distributions of responses across the five dimensions are presented in Supplementary Table S5.

The EQ VAS ranges from 0 to 100, with higher scores indicating better self-perceived health, and was used as the principal continuous health-related quality-of-life outcome. An EQ-5D utility index was not calculated because the use of a country-specific value set had not been specified in the analytical plan. Accordingly, the individual EQ-5D-3L dimensions were retained for descriptive supplementary analyses.

### 2.4. Statistical Analysis

#### 2.4.1. Descriptive and Unadjusted Analyses

Continuous variables are presented as means and standard deviations (SD) and categorical variables are presented as numbers and percentages. Participant characteristics were summarised for the overall study population and according to sex.

Continuous variables were compared between women and men using Welch’s *t*-test. Categorical variables were compared using Pearson’s chi-squared test. Exact tests were used when sparse cells made the asymptotic chi-squared approximation unsuitable. For multicategory cigarette-consumption and alcohol-consumption-frequency variables, exact *p* values were estimated using Monte Carlo simulation with 200,000 samples and fixed marginal totals.

Unadjusted differences between women and men are presented as mean differences (β), calculated as women minus men for continuous variables, and odds ratios (OR) comparing women with men for dichotomous variables, together with their 95% confidence intervals (CI) and *p* values. Fisher’s exact test was used for current smoking and alcohol consumption at least twice per week because of sparse cells.

The number and percentage of participants meeting each of the 14 MEDAS-14 criteria were calculated. Mean MEDAS-14 scores were compared across sociodemographic, lifestyle, and anthropometric characteristics using Welch’s *t*-test, Welch’s one-way analysis of variance, or the Kruskal–Wallis test, as appropriate. Welch’s one-way analysis of variance was used for comparisons involving more than two groups when parametric comparisons were appropriate, whereas the Kruskal–Wallis test was used for variables with sparse groups or relevant departures from parametric assumptions.

#### 2.4.2. Analyses According to Adherence Categories

Cardiometabolic, psychological, and health-related quality-of-life outcomes were compared across the exploratory low-, moderate-, and high-adherence categories using Welch’s one-way analysis of variance, which does not assume equal variances across groups.

Linear trends across adherence categories were assessed by assigning ordinal values of 0, 1, and 2 to the low-, moderate-, and high-adherence categories, respectively, and entering this ordinal variable as a continuous term in a linear regression model. Global *p* values represent overall differences among the three categories, whereas *p* values for trend assess whether there was evidence of a linear trend across the ordered categories.

These categorical comparisons were unadjusted and considered exploratory. For outcomes showing evidence of non-linearity in the fully adjusted models, additional multivariable models were fitted, with adherence category represented by indicator variables and low adherence as the reference category. These models included the same covariates as the corresponding fully adjusted continuous models and are reported in Supplementary Table S3.

#### 2.4.3. Multivariable Regression Models

Separate ordinary least-squares regression models were fitted for BMI, waist circumference, systolic blood pressure, diastolic blood pressure, fasting blood glucose, total cholesterol, Goldberg anxiety score, Goldberg depression score, and EQ VAS score. The MEDAS-14 score was entered as a continuous exposure. In the principal linear models, regression coefficients represent the adjusted mean difference in each outcome associated with a one-point increase in the MEDAS-14 score and are reported with 95% confidence intervals (CI) and two-sided *p* values.

Models for BMI and waist circumference were adjusted for age, sex, educational attainment, and physical activity. Models for systolic and diastolic blood pressure, fasting blood glucose, total cholesterol, anxiety, depression, and EQ VAS were additionally adjusted for BMI. Educational attainment and physical activity were entered as three-category variables and represented by indicator variables. BMI was not included as a covariate in the BMI or waist-circumference models to avoid adjustment for the outcome itself or for a closely related measure of adiposity.

To examine the influence of progressive covariate adjustment, crude models, models adjusted for age and sex, and fully adjusted models were estimated for each outcome (Supplementary Table S2).

#### 2.4.4. Model Diagnostics and Assessment of Non-Linearity

Ordinary least-squares model assumptions were assessed by examining residual distributions, residual-versus-fitted plots, heteroscedasticity, and influential observations. Heteroscedasticity-consistent HC3 standard errors were calculated as a sensitivity analysis.

Potential non-linearity was assessed by adding a quadratic term for the mean-centred MEDAS-14 score to each fully adjusted model. A statistically significant quadratic term (*p* < 0.05) was considered evidence of non-linearity. When evidence of non-linearity was identified, the corresponding linear coefficient was not interpreted as representing a constant difference across the entire MEDAS-14 score range.

For outcomes showing evidence of non-linearity, additional multivariable models were fitted using the exploratory adherence categories, with low adherence as the reference category. These models included the same covariates as the corresponding fully adjusted continuous models. Adjusted predictions derived from the quadratic models were calculated across the observed MEDAS-14 score range and plotted with their 95% confidence intervals (CIs) in Supplementary Figure S1. The distribution of participants across the observed MEDAS-14 scores was also displayed to illustrate the smaller number of observations at the extremes of the score distribution. These non-linearity analyses were considered exploratory.

#### 2.4.5. Sensitivity Analyses

Sensitivity analyses included: (1) fully adjusted models using HC3 heteroscedasticity-consistent standard errors; (2) models using an adherence score excluding the wine component, resulting in a 13-item score ranging from 0 to 13; (3) models additionally adjusted for current smoking; and (4) separate models additionally adjusted for a binary indicator of any versus no alcohol consumption. The complete sensitivity analyses, quadratic models described in Section 2.4.4, and adjusted categorical models for outcomes showing evidence of non-linearity are presented in Supplementary Table S3.

Exclusion of the wine component was conducted to examine whether the observed associations were driven by an item that awards one point for moderate alcohol consumption, given the continuing debate regarding the inclusion of wine consumption within Mediterranean diet adherence scores [16]. Models additionally adjusted for current smoking were considered sensitivity analyses because smoking may be associated with both Mediterranean diet adherence and the study outcomes but was not included in the principal adjustment set. Alcohol-consumption frequency was not included in the principal adjustment set because of conceptual overlap with the MEDAS-14 exposure through its wine-consumption component and because the available alcohol-use variables did not permit precise quantification of total alcohol intake. Residual confounding by alcohol consumption therefore could not be excluded. As an additional sensitivity analysis, the fully adjusted models were further adjusted for a binary indicator of any versus no alcohol consumption derived from the alcohol-consumption-frequency variable.

#### 2.4.6. Effect Modification by Sex

Potential effect modification by sex was evaluated using pooled multivariable models containing the continuous MEDAS-14 score, sex, and a MEDAS-14 score × sex interaction term. Sex-specific regression coefficients and formal interaction *p* values were estimated for each outcome (Supplementary Table S4).

Differences in the statistical significance of sex-specific coefficients were not interpreted as evidence of effect modification unless supported by the formal interaction test. Because interaction tests were performed for nine outcomes, false-discovery-rate-adjusted *q* values were calculated across the nine interaction *p* values using the Benjamini–Hochberg procedure [17]. These effect-modification analyses were considered exploratory.

#### 2.4.7. Missing Data, Statistical Significance, and Software

All 495 participants included in the analytical sample had complete information for the variables included in the principal regression models and for the individual EQ-5D-3L dimensions; therefore, no imputation was performed.

All statistical tests were two-sided. The principal analyses prioritised estimation of effect sizes and 95% confidence intervals. Because the same exposure was evaluated across nine principal outcomes, Benjamini–Hochberg false-discovery-rate-adjusted *q* values were additionally calculated across the nine principal-model *p* values to account for multiplicity and aid interpretation of the overall pattern of findings. A *p* value < 0.05 was used as the conventional threshold for the principal-model analyses, while the corresponding FDR-adjusted *q* values were considered alongside the effect estimates and confidence intervals. For the interaction analyses, both unadjusted interaction *p* values and Benjamini–Hochberg FDR-adjusted *q* values were reported. Given the exploratory nature of the secondary categorical, non-linear, interaction, and sensitivity analyses, results were interpreted according to the magnitude and precision of the estimates, consistency across model specifications, and multiplicity of comparisons rather than solely according to statistical significance.

All statistical analyses were performed using Python version 3.12.13 (Python Software Foundation, Wilmington, DE, USA), with pandas version 2.2.3, NumPy version 2.3.5, and SciPy version 1.17.0. Figures were generated using Matplotlib version 3.10.8.

### 2.5. Ethical Considerations

The study was conducted in accordance with the Declaration of Helsinki and was approved by the Navarra Research Ethics Committee (Comité de Ética de la Investigación con Medicamentos de Navarra, CEIm Navarra; protocol code PI_2024/22; approval date: 27 March 2024; favourable opinion issued on 28 May 2024). All participants received written information about the study and provided written informed consent before inclusion.

## 3. Results

### 3.1. Participant Characteristics

Of the 504 adults approached and assessed for participation, two were excluded because their cancer diagnoses met the exclusion criteria and seven declined to participate. The final analytical sample comprised 495 participants (Figure 1), including 290 women (58.6%) and 205 men (41.4%). The mean age was 48.3 ± 15.0 years, 257 participants (51.9%) had university education or higher, and 267 (53.9%) reported high physical activity. The mean BMI was 25.6 ± 4.9 kg/m^2^, and 103 participants (20.8%) were classified as having obesity. The mean MEDAS-14 score was 8.82 ± 2.03 points. Based on the exploratory adherence categories, 130 participants (26.3%) had low adherence, 224 (45.3%) had moderate adherence, and 141 (28.5%) had high adherence (Table 1).

Women were younger than men (46.2 ± 14.6 vs. 51.3 ± 15.0 years; *p* < 0.001) and had higher odds of having university education or higher (OR = 1.99; 95% CI: 1.38 to 2.86; *p* < 0.001) and of reporting high physical activity (OR = 2.74; 95% CI: 1.89 to 3.96; *p* < 0.001). Women also had lower mean BMI, waist circumference, systolic and diastolic blood pressure, and fasting blood glucose than men. There was no evidence of sex differences in obesity prevalence, total cholesterol, or EQ VAS score. Women reported higher anxiety and depression scores and had a mean MEDAS-14 score 1.31 points higher than men (95% CI: 0.97 to 1.65; *p* < 0.001) (Table 1 and Table 2).

### 3.2. Distribution and Correlates of the MEDAS-14 Score

The most frequently achieved MEDAS-14 components were the use of olive oil as the principal culinary fat (94.1%), consumption of less than one serving of red or processed meat per day (87.3%), consumption of less than one serving of butter, margarine, or cream per day (82.8%), and consumption of at least four tablespoons of olive oil per day (82.6%). The least frequently achieved components were consumption of at least seven glasses of wine per week (5.1%), at least three servings of legumes per week (32.5%), and at least three servings of fish or seafood per week (34.5%) (Table 3).

Mean MEDAS-14 scores differed according to sex, age, educational attainment, physical activity, BMI category, cigarette consumption, and alcohol-consumption frequency (all global *p* ≤ 0.008; Table 4). Women had higher mean scores than men (9.36 ± 2.02 vs. 8.05 ± 1.79; *p* < 0.001). Mean scores increased across age groups, from 8.45 ± 2.13 among participants aged 18–39 years to 9.20 ± 1.95 among those aged ≥60 years (global *p* = 0.008).

Participants with university education or higher had a higher mean score than those with secondary or vocational education (9.12 ± 1.91 vs. 8.29 ± 2.03), although the overall educational pattern was not monotonic. Participants reporting high physical activity had the highest mean score (9.45 ± 1.88), whereas those reporting moderate physical activity had the lowest mean score (7.81 ± 1.69; global *p* < 0.001).

Among participants with normal weight, overweight, and obesity, mean MEDAS-14 scores were progressively lower across these categories (9.22 ± 1.72, 8.60 ± 2.26, and 8.04 ± 2.27, respectively; global *p* < 0.001). MEDAS-14 scores also differed across cigarette-consumption categories and alcohol-consumption-frequency categories (both global *p* < 0.001), although the pattern across alcohol-consumption categories was not monotonic.

### 3.3. Outcomes According to Exploratory Adherence Categories

Anthropometric, cardiometabolic, psychological, and health-related quality-of-life outcomes were compared across the exploratory MEDAS-14 adherence categories (Table 5). Mean BMI was 28.4 ± 4.5 kg/m^2^ in the low-adherence category, 24.8 ± 5.0 kg/m^2^ in the moderate-adherence category, and 24.5 ± 4.2 kg/m^2^ in the high-adherence category (global *p* < 0.001; *p* for trend < 0.001). Waist circumference showed a similar pattern, with mean values of 94.5 ± 10.8 cm, 87.1 ± 13.2 cm, and 85.1 ± 12.5 cm, respectively (global *p* < 0.001; *p* for trend < 0.001).

Mean systolic blood pressure was 127.0 ± 15.9, 126.4 ± 16.1, and 121.5 ± 14.5 mmHg in the low-, moderate-, and high-adherence categories, respectively (global *p* = 0.003; *p* for trend = 0.003). The corresponding mean diastolic blood pressure values were 80.4 ± 8.6, 77.5 ± 10.0, and 75.8 ± 9.4 mmHg (global *p* < 0.001; *p* for trend < 0.001).

Mean fasting blood glucose concentrations were 88.0 ± 13.9, 87.3 ± 17.7, and 82.7 ± 8.5 mg/dL across the low-, moderate-, and high-adherence categories, respectively (global *p* < 0.001; *p* for trend = 0.002). Total cholesterol differed across the categories (201.2 ± 47.0, 192.4 ± 32.5, and 201.8 ± 41.7 mg/dL, respectively; global *p* = 0.031)**,** but there was no evidence of a linear trend (*p* for trend = 0.845).

Mean Goldberg anxiety scores were 4.78 ± 2.20, 3.32 ± 2.61, and 3.30 ± 2.84 in the low-, moderate-, and high-adherence categories, respectively (global *p* < 0.001; *p* for trend < 0.001). Goldberg depression scores also differed across categories (2.70 ± 2.35, 1.99 ± 2.26, and 2.62 ± 2.75, respectively; global *p* = 0.008)**;** however, the observed pattern was non-monotonic, and there was no evidence of a linear trend (*p* for trend = 0.845).

Mean EQ VAS scores were 70.0 ± 12.7 in the low-adherence category, 73.4 ± 15.1 in the moderate-adherence category, and 75.8 ± 13.8 in the high-adherence category (global *p* = 0.002; *p* for trend < 0.001). All categorical comparisons were unadjusted and exploratory.

**Table 5:** Cardiometabolic, psychological, and health-related quality-of-life outcomes according to exploratory MEDAS-14 adherence categories.

Outcome	Low Adherence, ≤7 (*n* = 130)	Moderate Adherence, 8–9 (*n* = 224)	High Adherence, ≥10 (*n* = 141)	Global *p* Value	*p* for Trend
Anthropometric outcomes					
Body mass index, kg/m^2^	28.4 ± 4.5	24.8 ± 5.0	24.5 ± 4.2	<0.001	<0.001
Waist circumference, cm	94.5 ± 10.8	87.1 ± 13.2	85.1 ± 12.5	<0.001	<0.001
Clinical outcomes					
Systolic blood pressure, mmHg	127.0 ± 15.9	126.4 ± 16.1	121.5 ± 14.5	0.003	0.003
Diastolic blood pressure, mmHg	80.4 ± 8.6	77.5 ± 10.0	75.8 ± 9.4	<0.001	<0.001
Fasting blood glucose, mg/dL	88.0 ± 13.9	87.3 ± 17.7	82.7 ± 8.5	<0.001	0.002
Total cholesterol, mg/dL	201.2 ± 47.0	192.4 ± 32.5	201.8 ± 41.7	0.031	0.845
Psychological outcomes					
Goldberg anxiety score	4.78 ± 2.20	3.32 ± 2.61	3.30 ± 2.84	<0.001	<0.001
Goldberg depression score	2.70 ± 2.35	1.99 ± 2.26	2.62 ± 2.75	0.008	0.845
Health-related quality of life					
EuroQol VAS score	70.0 ± 12.7	73.4 ± 15.1	75.8 ± 13.8	0.002	<0.001

Note: Values are presented as mean ± standard deviation. Global *p* values were obtained using Welch’s one-way analysis of variance, which does not assume equal variances. Linear trend was evaluated by entering the ordered adherence categories as a single ordinal variable coded 0, 1, and 2. These comparisons were unadjusted and were considered exploratory. The adherence categories should not be interpreted as validated clinical thresholds. All tests were two-sided. MEDAS-14: 14-item Mediterranean Diet Adherence Screener; VAS: visual analogue scale.

### 3.4. Adjusted Associations Between MEDAS-14 and Study Outcomes

In the fully adjusted linear models, the MEDAS-14 score was inversely associated with BMI (β = −0.299 kg/m^2^ per one-point increase; 95% CI: −0.521 to −0.077; *p* = 0.009) (Table 6). The estimated linear association with waist circumference was also inverse, although its confidence interval included the null value (β = −0.475 cm per point; 95% CI: −1.046 to 0.095; *p* = 0.102). Evidence of non-linearity was identified for both anthropometric outcomes; therefore, these linear coefficients should not be interpreted as constant differences across the entire MEDAS-14 score range.

After adjustment, higher MEDAS-14 scores were associated with lower systolic blood pressure (β = −1.056 mmHg per point; 95% CI: −1.774 to −0.338; *p* = 0.004), lower diastolic blood pressure (β = −0.838 mmHg per point; 95% CI: −1.261 to −0.415; *p* < 0.001), and lower fasting blood glucose concentrations (β = −0.745 mg/dL per point; 95% CI: −1.365 to −0.125; *p* = 0.019).

There was no evidence of an adjusted linear association between the MEDAS-14 score and total cholesterol (β = −0.823 mg/dL per point; 95% CI: −2.621 to 0.975; *p* = 0.369). However, evidence of non-linearity was identified for total cholesterol; consequently, the corresponding linear coefficient should be interpreted cautiously.

Higher MEDAS-14 scores were associated with lower Goldberg anxiety scores (β = −0.249 points per one-point increase; 95% CI: −0.368 to −0.130; *p* < 0.001). There was no evidence of an adjusted association with the Goldberg depression score (β = 0.033; 95% CI: −0.086 to 0.152; *p* = 0.590). Higher MEDAS-14 scores were also associated with better self-perceived health, with an adjusted 1.224-point higher EQ VAS score per one-point increase in the MEDAS-14 score (β = 1.224; 95% CI: 0.570 to 1.878; *p* < 0.001).

**Table 6:** Adjusted associations of the MEDAS-14 score with cardiometabolic, psychological, and health-related quality-of-life outcomes.

Outcome	Adjusted β per One-Point Increase in MEDAS-14	95% CI	*p* Value
Anthropometric outcomes			
Body mass index, kg/m^2 ac^	−0.299	−0.521 to −0.077	0.009
Waist circumference, cm ^ac^	−0.475	−1.046 to 0.095	0.102
Clinical outcomes			
Systolic blood pressure, mmHg ^b^	−1.056	−1.774 to −0.338	0.004
Diastolic blood pressure, mmHg ^b^	−0.838	−1.261 to −0.415	<0.001
Fasting blood glucose, mg/dL ^b^	−0.745	−1.365 to −0.125	0.019
Total cholesterol, mg/dL ^bc^	−0.823	−2.621 to 0.975	0.369
Psychological outcomes			
Goldberg anxiety score ^b^	−0.249	−0.368 to −0.130	<0.001
Goldberg depression score ^b^	0.033	−0.086 to 0.152	0.590
Health-related quality of life			
EQ VAS score ^b^	1.224	0.570 to 1.878	<0.001

Note: Regression coefficients were estimated using separate multivariable ordinary least-squares regression models for each outcome. Each coefficient represents the adjusted mean difference in the corresponding outcome associated with a one-point increase in the MEDAS-14 score. Negative coefficients indicate lower outcome values with higher MEDAS-14 scores, whereas positive coefficients indicate higher outcome values. All tests were two-sided. BMI: body mass index; CI: confidence interval; EQ VAS: EuroQol visual analogue scale; MEDAS-14: 14-item Mediterranean Diet Adherence Screener. ^a^ Adjusted for age, sex, educational attainment, and physical activity. BMI was not included as a covariate in the BMI or waist-circumference models to avoid adjustment for the outcome itself or for a closely related measure of adiposity. ^b^ Adjusted for age, sex, educational attainment, physical activity, and BMI. Educational attainment and physical activity were entered as categorical variables. ^c^ Evidence of non-linearity was detected for this outcome. Therefore, the linear coefficient should not be interpreted as a constant change across the entire MEDAS-14 score range. Adjusted categorical and non-linear analyses are presented in Supplementary Table S3 and Supplementary Figure S1.

### 3.5. Progressive Models, Sensitivity Analyses, and Non-Linearity

The associations with BMI, systolic and diastolic blood pressure, fasting blood glucose, Goldberg anxiety score, and EQ VAS remained statistically significant after progressive adjustment for age, sex, educational attainment, physical activity, and, when appropriate, BMI (Supplementary Table S2). Covariate adjustment substantially attenuated the associations with BMI and waist circumference. Total cholesterol and Goldberg depression score were not consistently associated with the continuous MEDAS-14 score across the progressive models.

The associations with systolic and diastolic blood pressure, fasting blood glucose, Goldberg anxiety score, and EQ VAS remained statistically significant when HC3 heteroscedasticity-consistent standard errors were used, when the wine component was excluded from the adherence score, and after additional adjustment for current smoking (Supplementary Table S3). There was no evidence of an association between the MEDAS-14 score and the Goldberg depression score in any of these sensitivity analyses.

The principal associations were also materially unchanged after additional adjustment for any versus no alcohol consumption. Associations with BMI, systolic and diastolic blood pressure, fasting blood glucose, Goldberg anxiety score, and EQ VAS remained statistically significant, whereas those for waist circumference, total cholesterol, and Goldberg depression score remained non-significant (Supplementary Table S3).

Benjamini–Hochberg false-discovery-rate correction was additionally applied across the nine principal outcome models. The overall pattern of findings was unchanged after FDR correction: the associations with BMI, systolic and diastolic blood pressure, fasting blood glucose, Goldberg anxiety score, and EQ VAS retained *q* values < 0.05, whereas those for waist circumference, total cholesterol, and Goldberg depression score did not (Supplementary Table S3).

The inverse linear associations with BMI and waist circumference were attenuated after additional adjustment for current smoking. The smoking-adjusted coefficient was −0.169 kg/m^2^ for BMI (95% CI: −0.395 to 0.058; *p* = 0.144) and −0.338 cm for waist circumference (95% CI: −0.929 to 0.252; *p* = 0.260).

Evidence of non-linearity was identified for BMI, waist circumference, and total cholesterol, with statistically significant quadratic terms for BMI (*p* < 0.001), waist circumference (*p* = 0.002), and total cholesterol (*p* < 0.001). In the adjusted categorical models, both the moderate- and high-adherence categories were associated with lower BMI and waist circumference compared with the low-adherence category. The adjusted mean BMI differences were −2.949 kg/m^2^ for moderate versus low adherence (95% CI: −3.980 to −1.917; *p* < 0.001) and −2.510 kg/m^2^ for high versus low adherence (95% CI: −3.730 to −1.290; *p* < 0.001). The corresponding adjusted differences in waist circumference were −5.461 cm (95% CI: −8.137 to −2.785; *p* < 0.001) and −4.992 cm (95% CI: −8.157 to −1.826; *p* = 0.002), respectively.

Total cholesterol displayed a non-monotonic pattern. Compared with low adherence, the adjusted mean difference was −19.316 mg/dL for moderate adherence (95% CI: −27.874 to −10.759; *p* < 0.001) and −7.901 mg/dL for high adherence (95% CI: −17.872 to 2.070; *p* = 0.120). The continuous linear association with total cholesterol was not statistically significant in the principal model, the HC3 analysis, the analysis excluding the wine component, or the models additionally adjusted for current smoking or any versus no alcohol consumption. The cholesterol findings were therefore considered exploratory and were interpreted primarily in relation to the observed non-linear pattern.

The fitted quadratic curves for BMI, waist circumference, and total cholesterol showed U-shaped patterns, with the lowest fitted predicted values occurring at MEDAS-14 scores of approximately 9–10 points (Supplementary Figure S1). Confidence intervals widened toward the extremes of the observed MEDAS-14 score distribution, where fewer observations were available. These non-linear patterns were considered exploratory.

### 3.6. Effect Modification by Sex

After false-discovery-rate correction, evidence of interaction by sex remained for diastolic blood pressure and EQ VAS (Supplementary Table S4). For diastolic blood pressure, the adjusted association with the MEDAS-14 score was more negative among men (β = −1.782 mmHg per one-point increase; 95% CI: −2.467 to −1.096; *p* < 0.001) than among women (β = −0.383 mmHg per one-point increase; 95% CI: −0.877 to 0.110; *p* = 0.128). The formal interaction test indicated that the association differed by sex (interaction *p* < 0.001; FDR-adjusted *q* = 0.003).

For EQ VAS, the sex-specific coefficient was positive among women (β = 1.942 points per one-point increase in the MEDAS-14 score; 95% CI: 1.179 to 2.705; *p* < 0.001), whereas there was no evidence of an association among men (β = −0.267 points; 95% CI: −1.327 to 0.793; *p* = 0.621). The formal interaction test indicated that the association differed by sex (interaction *p* < 0.001; FDR-adjusted *q* = 0.003).

A nominal interaction was observed for waist circumference (interaction *p* = 0.039), but this did not remain statistically significant after false-discovery-rate correction (*q* = 0.102). The interactions for fasting blood glucose (interaction *p* = 0.051; *q* = 0.102) and Goldberg anxiety score (interaction *p* = 0.056; *q* = 0.102) were also not statistically significant after FDR correction. There was no evidence of interaction for BMI, systolic blood pressure, total cholesterol, or Goldberg depression score. All interaction and sex-specific analyses were considered exploratory.

### 3.7. Additional Descriptive Findings

Across the individual EQ-5D-3L dimensions, most participants reported no problems with mobility (87.7%), self-care (94.7%), or usual activities (91.7%). Moderate or extreme pain/discomfort was reported by 196 participants (39.6%), and moderate or extreme anxiety/depression was reported by 266 participants (53.7%) (Supplementary Table S5).

Overall, 438 participants (88.5%) reported no current cigarette consumption, and 98 (19.8%) reported never consuming alcohol. Premature cardiovascular disease in a first-degree relative was reported by 64 participants (12.9%). The number of alcoholic drinks consumed on a typical drinking day was retained in its original categorical form, with participants who reported never consuming alcohol coded as 0 (not applicable). Because these response categories represented ranges rather than exact quantities, this variable was not used to derive a quantitative measure of total alcohol intake or included as a quantitative covariate in the regression models (Supplementary Table S6). A separate binary indicator of any versus no alcohol consumption, derived from the alcohol-consumption-frequency variable, was used only in the sensitivity analysis described above.

## 4. Discussion

In this cross-sectional study of 495 adults attending primary care centres in Navarra, higher Mediterranean diet adherence, as assessed using the MEDAS-14 score, was associated with several cardiometabolic, psychological, and self-perceived health outcomes. After adjustment for age, sex, educational attainment, physical activity, and, when appropriate, BMI, higher MEDAS-14 scores were associated with lower systolic and diastolic blood pressure, lower fasting blood glucose, fewer anxiety symptoms, and higher EQ VAS scores. There was no evidence of an adjusted association with the Goldberg depression score or of an adjusted linear association with total cholesterol. Although the principal linear model showed an inverse association with BMI, evidence of non-linearity was identified for BMI, waist circumference, and total cholesterol, indicating that these outcomes could not be adequately characterised by a single constant per-point coefficient across the entire MEDAS-14 score range.

The associations with systolic and diastolic blood pressure, fasting blood glucose, Goldberg anxiety score, and EQ VAS were generally consistent across the progressive models and sensitivity analyses using HC3 heteroscedasticity-consistent standard errors, a 13-item adherence score excluding the wine component, and additional adjustment for current smoking and any versus no alcohol consumption. The overall pattern of principal findings was also unchanged after Benjamini–Hochberg false-discovery-rate correction across the nine principal outcome models. In contrast, the associations involving BMI and waist circumference were attenuated after additional adjustment for current smoking, while total cholesterol showed no statistically significant continuous linear association across the principal and sensitivity models. These differences in robustness, together with the evidence of non-linearity for BMI, waist circumference, and total cholesterol, should be considered when interpreting the relative strength of the findings.

Exploratory interaction analyses showed evidence of interaction by sex for diastolic blood pressure and EQ VAS after false-discovery-rate correction. The inverse association between MEDAS-14 and diastolic blood pressure was more pronounced among men, whereas the positive association with EQ VAS was more pronounced among women. Because these analyses were exploratory and based on formal interaction testing across multiple outcomes, these findings should be regarded as hypothesis-generating and warrant replication in independent studies.

The inverse associations between Mediterranean diet adherence and systolic and diastolic blood pressure are consistent with evidence from systematic reviews and dietary intervention trials [4,18]. A meta-analysis of 35 randomised controlled trials involving 13,943 participants found that Mediterranean dietary interventions produced relatively small reductions in systolic and diastolic blood pressure compared with control diets, with pooled mean differences of −1.5 and −0.9 mmHg, respectively [18]. These estimates are broadly similar in magnitude to the adjusted per-point differences observed in MEDINAV, although direct comparisons should be made cautiously because the meta-analysis evaluated dietary interventions, whereas the present study examined cross-sectional associations with a continuous adherence score.

Evidence from the PREDIMED trial further showed a lower incidence of major cardiovascular events among adults at high cardiovascular risk assigned to Mediterranean diets supplemented with extra-virgin olive oil or nuts than among those assigned to receive advice to reduce dietary fat [19]. However, PREDIMED evaluated a dietary intervention and clinical cardiovascular events in a high-risk population, whereas MEDINAV examined cross-sectional associations with cardiovascular risk factors in a primary care population.

The Mediterranean dietary pattern provides fibre, unsaturated fatty acids, polyphenols, potassium, and other bioactive compounds that may contribute to endothelial function, inflammatory and oxidative processes, and regulation of vascular tone [3,4]. Inflammatory pathways are also central to cardiovascular pathophysiology, including the response to myocardial injury and subsequent cardiac remodelling [20]. In the present study, each one-point higher MEDAS-14 score was associated with approximately 1.06 mmHg lower systolic blood pressure and 0.84 mmHg lower diastolic blood pressure after adjustment. These coefficients represent adjusted mean differences and should not be interpreted as causal effects at the individual level. Although differences of this magnitude across several points of the adherence score may be potentially relevant at the population level, the cross-sectional design does not establish whether greater dietary adherence preceded lower blood pressure values.

After adjustment, higher MEDAS-14 scores were also associated with lower fasting blood glucose concentrations, with an adjusted mean difference of −0.745 mg/dL per one-point higher MEDAS-14 score. This finding is consistent with recent meta-analytic evidence linking greater Mediterranean diet adherence to a lower risk of type 2 diabetes [5] and with a meta-analysis of randomised controlled trials reporting improvements in fasting plasma glucose and glycated haemoglobin among adults with established type 2 diabetes assigned to Mediterranean dietary interventions [21]. However, the latter evidence was derived from a clinical population with diabetes and is therefore not directly comparable with the present primary care sample. Potential explanatory mechanisms include higher fibre intake, improved dietary fat quality, lower glycaemic load, and favourable effects on insulin sensitivity and body weight [3,5]. The persistence of the association after adjustment for BMI indicates that the observed association was not fully accounted for by BMI in the fitted model. Nevertheless, diabetes status, glucose-lowering medication use, and other metabolic factors were not included in the adjustment set and should be considered when interpreting this finding.

Insulin resistance may represent an additional pathway linking Mediterranean diet adherence with the cardiometabolic and psychological outcomes examined in the present study. Mediterranean dietary patterns, characterised by a high intake of plant-based foods, fibre, unsaturated fatty acids, and bioactive compounds, have been associated with more favourable insulin sensitivity and glycaemic regulation [3,5,21]. Because insulin resistance is closely related to endothelial dysfunction, increased sympathetic activity, inflammation, and other metabolic disturbances, improved insulin sensitivity could potentially contribute to more favourable blood pressure profiles. Insulin resistance and related inflammatory and metabolic pathways have also been implicated in psychological symptoms and may therefore represent one possible pathway underlying the inverse association observed between MEDAS-14 adherence and anxiety symptoms. However, fasting insulin concentrations and direct measures of insulin resistance, such as HOMA-IR, were not available in MEDINAV. Therefore, insulin resistance could not be evaluated as a mediator of the observed associations, and this mechanistic interpretation should be considered hypothesis-generating.

The absence of a statistically significant adjusted linear association with total cholesterol also warrants consideration. In the principal model, the adjusted coefficient was −0.823 mg/dL per one-point higher MEDAS-14 score, with a confidence interval including the null value (*p* = 0.369). Recent meta-analytic evidence has shown inconclusive associations between Mediterranean diet adherence and hypercholesterolaemia or hypertriglyceridaemia [5]. Similarly, a meta-analysis of randomised controlled trials among adults with type 2 diabetes found improvements in blood pressure and glycaemic control but no statistically significant reduction in total cholesterol [21]. Mediterranean dietary patterns may have more apparent associations with specific lipoprotein fractions, triglycerides, lipid oxidation, or overall cardiovascular risk than with total cholesterol alone [3,4].

In MEDINAV, the continuous linear association with total cholesterol was also not statistically significant in the HC3 analysis, the analysis excluding the wine component, or the models additionally adjusted for current smoking or any versus no alcohol consumption. However, evidence of non-linearity was identified, with a statistically significant quadratic term (*p* < 0.001). Accordingly, these findings do not support a simple linear dose–response relationship between MEDAS-14 and total cholesterol and should be interpreted as exploratory. The database did not include a complete lipid profile, limiting the ability to examine potentially more informative lipid outcomes.

Moderate and high MEDAS-14 adherence were associated with lower adjusted BMI and waist circumference compared with low adherence. Previous umbrella-review and meta-analytic evidence has associated greater Mediterranean diet adherence with more favourable body-weight and central-adiposity outcomes [4,5]. More specifically, a systematic review and meta-analysis of 26 randomised controlled trials involving 10,352 adults with overweight or obesity found modest reductions in BMI and waist circumference following Mediterranean dietary interventions, with pooled mean differences of −0.61 kg/m^2^ and −2.48 cm, respectively [22]. However, the quality of evidence was low or very low for most outcomes, and the population was limited to adults with overweight or obesity. Direct comparisons with the broader primary care population included in MEDINAV should therefore be made cautiously.

In MEDINAV, the principal linear model showed an inverse association between MEDAS-14 and BMI, whereas the corresponding linear association with waist circumference was not statistically significant after full adjustment. Both associations were further attenuated after additional adjustment for current smoking, with smoking-adjusted coefficients of −0.169 kg/m^2^ for BMI (*p* = 0.144) and −0.338 cm for waist circumference (*p* = 0.260). This attenuation is compatible with the possibility that residual lifestyle confounding may partly account for the observed relationships. Moreover, evidence of non-linearity for both outcomes indicates that the corresponding linear coefficients should not be interpreted as constant differences across the entire MEDAS-14 score range.

Statistically significant quadratic terms were identified for BMI (*p* < 0.001), waist circumference (*p* = 0.002), and total cholesterol (*p* < 0.001). The adjusted quadratic curves showed U-shaped patterns, with the lowest adjusted predicted values occurring at MEDAS-14 scores of approximately 9–10 and some attenuation of the fitted differences at higher observed scores. In practical terms, this indicates that the associations were not adequately represented by a constant linear slope across the observed MEDAS-14 range. However, these patterns should not be interpreted as indicating that a MEDAS-14 score of approximately 9–10 is clinically optimal, that higher observed MEDAS-14 scores are necessarily associated with further reductions in these outcomes, or that higher adherence is detrimental. Confidence intervals widened toward the extremes of the observed score distribution, where fewer observations were available, and quadratic models impose a specific functional form that may not fully capture a more complex underlying association. These findings should therefore be regarded as exploratory and warrant confirmation in larger samples using more flexible modelling approaches, such as restricted cubic splines. This interpretation is consistent with the supplementary non-linearity analyses, which showed significant quadratic terms for BMI, waist circumference, and total cholesterol.

Reverse causation or confounding by indication may also contribute to the observed non-linear anthropometric patterns. Participants with overweight, obesity, or other cardiometabolic risk factors may have previously received dietary advice or modified their eating habits in response to their health status. In a cross-sectional analysis, such behavioural changes could attenuate or distort the observed relationship between current MEDAS-14 adherence and adiposity, and could contribute to an apparent non-linear pattern. Because previous dietary advice and the timing of dietary changes in relation to weight status were not assessed, this possibility cannot be evaluated directly in the present study.

The non-monotonic total-cholesterol findings reinforce this caution. In the adjusted categorical analysis, total cholesterol was lower in the moderate-adherence category than in the low-adherence category (adjusted mean difference −19.316 mg/dL; 95% CI: −27.874 to −10.759; *p* < 0.001), whereas the difference between the high- and low-adherence categories was smaller and not statistically significant (−7.901 mg/dL; 95% CI: −17.872 to 2.070; *p* = 0.120). In addition, the continuous linear association with total cholesterol was not statistically significant in the principal model, the HC3 analysis, the analysis excluding the wine component, or the models additionally adjusted for current smoking or any versus no alcohol consumption. One possible contributor to the non-monotonic cholesterol pattern is the wine component of the MEDAS-14, because alcohol consumption may influence lipid concentrations. However, only 25 participants (5.1%) met the MEDAS-14 wine criterion, and the continuous association with total cholesterol remained non-significant when the wine component was removed from the adherence score. This suggests that the observed pattern is unlikely to be explained solely by the wine component. Other unmeasured dietary characteristics, underlying dyslipidaemia, medication use, or residual confounding may also have contributed. Taken together, these findings do not support a simple or stable linear dose–response relationship between the MEDAS-14 score and total cholesterol and should be interpreted as exploratory.

Higher MEDAS-14 adherence was consistently associated with fewer anxiety symptoms. This association persisted after adjustment for sociodemographic characteristics, physical activity, BMI, and current smoking, after exclusion of the wine component from the adherence score, and after additional adjustment for any versus no alcohol consumption. Previous research has linked greater Mediterranean diet adherence with better mental health outcomes, including fewer anxiety symptoms, although the strength and certainty of the evidence vary according to the population, outcome definition, dietary assessment method, and study design [6].

Several biological and behavioural pathways could contribute to the observed association between diet and anxiety. Potential mechanisms include lower systemic inflammation and oxidative stress, more favourable vascular and metabolic profiles, greater intake of nutrients involved in neurotransmitter synthesis, and modulation of the gut–brain axis [3,6]. Nevertheless, the present findings cannot establish the direction of the association. Participants with fewer anxiety symptoms may find it easier to maintain healthier eating behaviours, whereas anxiety may also influence appetite, food choices, and adherence to dietary recommendations.

There was no evidence of an adjusted association between the MEDAS-14 score and depressive symptoms. Depression scores displayed a non-monotonic pattern across the exploratory adherence categories, and there was no evidence of a linear trend across these categories. The continuous MEDAS-14 score also remained unassociated with the Goldberg depression score in the fully adjusted and sensitivity models. This differs from systematic-review and meta-analytic evidence linking healthier dietary patterns with a lower risk of depressive outcomes [6,23]. A meta-analysis of observational studies found that the highest category of Mediterranean diet adherence was associated with a lower risk of incident depression than the lowest category [23]. However, the included studies varied in their dietary assessment methods, definitions of depression, study populations, and analytical approaches. Moreover, observational evidence cannot exclude residual confounding or establish causality.

Prospective evidence from the Spanish SUN cohort also found that greater adherence to Mediterranean and other healthy dietary patterns was associated with a lower incidence of depression [24]. However, the dose–response relationship was non-linear, with the greatest difference observed between low and moderate adherence and little additional benefit at higher adherence levels [24]. Direct comparisons with MEDINAV should be made cautiously because the SUN study used a nine-component Mediterranean Diet Score and defined incident depression according to a physician-reported diagnosis or initiation of antidepressant treatment. In contrast, MEDINAV used the MEDAS-14 and assessed current symptoms using the Goldberg depression subscale. The Goldberg subscales are screening instruments and should not be interpreted as providing clinical psychiatric diagnoses.

Higher MEDAS-14 adherence was also associated with higher EQ VAS scores, indicating better self-perceived health. Each one-point higher MEDAS-14 score was associated with an adjusted 1.224-point higher EQ VAS score, and this association remained statistically significant across the sensitivity analyses, including additional adjustment for current smoking and any versus no alcohol consumption. Systematic-review evidence has generally linked greater Mediterranean diet adherence with better health-related quality of life in adult populations [7]. However, findings from individual prospective studies have not been entirely consistent. In two Spanish cohorts of community-dwelling older adults, greater Mediterranean diet adherence was not associated with a clinically relevant improvement in health-related quality of life, although a small improvement in the physical component was observed in one cohort [25].

Differences between that study and MEDINAV should be considered. Pérez-Tasigchana et al. included adults aged ≥60 years and assessed health-related quality of life using the SF-36 or SF-12, whereas MEDINAV included a broader adult age range and used the EQ VAS [25]. The clinical relevance of the EQ VAS coefficient observed in MEDINAV cannot be established because no population-specific minimally important difference was prespecified. Moreover, the EQ VAS provides a global assessment of perceived health and may reflect physical health, psychological well-being, socioeconomic circumstances, and other unmeasured factors.

In the unadjusted descriptive analyses, women had higher mean MEDAS-14 scores than men and were more frequently classified in the exploratory high-adherence category. Mean adherence scores also differed across age, educational-attainment, physical-activity, cigarette-consumption, and alcohol-consumption-frequency categories. Older participants had progressively higher mean MEDAS-14 scores across age groups, and participants reporting high physical activity had the highest mean adherence score. Women were also substantially more frequently represented in the high-adherence category than men (37.9% vs. 15.1%). These findings indicate that Mediterranean diet adherence was patterned according to several sociodemographic and lifestyle characteristics in this sample. However, because these comparisons were unadjusted, they should not be interpreted as identifying independent determinants of adherence.

The relationships with educational attainment and physical activity were not monotonic. Participants with secondary or vocational education had lower mean MEDAS-14 scores than those in both the basic-education and university-or-higher groups, whereas participants reporting moderate physical activity had lower mean scores than those in the low/inactive and high-activity categories. These patterns may reflect differences in age, occupation, socioeconomic circumstances, cultural background, or the classification and self-reporting of physical activity. They should therefore not be interpreted as simple linear social or behavioural gradients.

MEDAS-14 scores also differed across cigarette-consumption and alcohol-consumption-frequency categories, although neither pattern was strictly monotonic. The relationship with alcohol-consumption frequency should be interpreted cautiously because the MEDAS-14 includes a component awarding one point when the wine-consumption criterion is met, creating partial conceptual overlap between the adherence score and alcohol-related behaviour. Nevertheless, the principal associations with systolic and diastolic blood pressure, fasting blood glucose, anxiety symptoms, and EQ VAS remained statistically significant when the wine component was excluded from the adherence score. The overall pattern of principal associations was also materially unchanged after additional adjustment for any versus no alcohol consumption.

The clustering of Mediterranean diet adherence with physical activity, smoking behaviour, educational attainment, alcohol consumption, and other participant characteristics is compatible with a broader pattern of health-related behaviours rather than an isolated dietary behaviour. Although the multivariable outcome models were adjusted for age, sex, educational attainment, physical activity, and BMI when appropriate, and current smoking and any versus no alcohol consumption were additionally considered in sensitivity analyses, residual and unmeasured confounding cannot be excluded.

Exploratory interaction analyses showed evidence of interaction by sex for diastolic blood pressure and EQ VAS after false-discovery-rate correction. The inverse association between the MEDAS-14 score and diastolic blood pressure was more pronounced among men than among women, whereas the positive association between MEDAS-14 and EQ VAS was more pronounced among women. Both formal interaction tests remained statistically significant after false-discovery-rate correction (*q* = 0.003 for both outcomes).

These findings may reflect biological, behavioural, or socially patterned differences in dietary habits, baseline cardiovascular profiles, psychosocial circumstances, or the reporting of perceived health. However, the study was not primarily designed to evaluate interaction effects, and the sex-specific estimates were based on smaller subgroups than the principal analyses. A nominal interaction was also observed for waist circumference (*p* = 0.039), but this did not remain statistically significant after correction for multiple testing (*q* = 0.102). The interaction for fasting blood glucose was close to, but did not meet, the conventional threshold for statistical significance (*p* = 0.051; *q* = 0.102). The findings for diastolic blood pressure and EQ VAS should therefore be regarded as hypothesis-generating and warrant replication in independent studies.

Previous randomised intervention research has reported sex-dependent glycaemic responses to Mediterranean dietary patterns with different glycaemic indices. In the MEDGI-Carb trial, which included 156 adults at elevated risk of type 2 diabetes, women showed higher postprandial glucose concentrations with the high-glycaemic-index Mediterranean diet than with the low-glycaemic-index diet, whereas no corresponding difference was observed among men; the formal sex × diet interaction was statistically significant [26]. However, that trial compared two Mediterranean dietary interventions differing principally in their major sources of starch and evaluated eight-hour glucose and insulin profiles. It therefore does not directly explain the interactions observed in MEDINAV for diastolic blood pressure or EQ VAS. Nevertheless, it provides some support for the broader hypothesis that responses to specific characteristics of Mediterranean dietary patterns may differ by sex.

The findings suggest that brief assessment of Mediterranean diet adherence may have potential value in primary care as a means of identifying dietary behaviours that could be addressed during preventive counselling. In MEDINAV, relatively few participants met the MEDAS-14 criteria for legumes and fish or seafood, and fewer than half met the fruit-consumption criterion. A systematic review of international clinical guidelines identified dietary interventions for primary cardiovascular prevention that may be applicable in primary care and community settings, although detailed implementation strategies were generally lacking [27]. Furthermore, a cluster-randomised trial conducted in 26 Spanish primary healthcare centres showed that a multilevel intervention delivered by primary care professionals produced a modest but statistically significant improvement in Mediterranean diet adherence compared with brief usual-care advice [28]. Together, this external evidence supports the feasibility of assessing and promoting Mediterranean dietary behaviours in routine primary care, although MEDINAV itself did not evaluate an intervention or implementation strategy.

The MEDAS-14 wine criterion was met by only 25 participants (5.1%). This finding should not be interpreted as indicating that alcohol consumption should be initiated or increased. Importantly, the associations with systolic and diastolic blood pressure, fasting blood glucose, Goldberg anxiety score, and EQ VAS remained statistically significant when the wine component was excluded from the adherence score. The overall pattern of principal associations was also materially unchanged after additional adjustment for any versus no alcohol consumption. These sensitivity analyses indicate that the principal findings were not materially dependent on awarding one point for meeting the wine-consumption criterion and are particularly relevant given the continuing debate regarding the inclusion of alcohol consumption within Mediterranean diet adherence scores [16].

From a practical perspective, dietary counselling in similar primary care populations could place particular emphasis on components for which adherence was relatively low in MEDINAV, including legumes, fish or seafood, and fruit. Other components of the Mediterranean dietary pattern, such as vegetables, nuts, olive oil, lower consumption of red and processed meat, commercial pastries, and sugar-sweetened beverages, may also be addressed according to individual dietary needs. However, MEDINAV did not evaluate the effects of dietary counselling or dietary change, and its cross-sectional findings do not demonstrate that increasing an individual’s MEDAS-14 score would produce subsequent improvements in cardiometabolic, psychological, or health-related quality-of-life outcomes.

Prospective studies are needed to establish temporality and determine whether changes in Mediterranean diet adherence precede subsequent changes in blood pressure, fasting blood glucose, psychological symptoms, and health-related quality of life. Intervention and implementation studies should also evaluate how brief dietary assessment and counselling strategies can be integrated effectively into routine primary care and whether improvements in adherence are sustained over time. Larger samples would permit more reliable assessment of the non-linear associations observed for BMI, waist circumference, and total cholesterol and of the exploratory interactions by sex. Future studies should incorporate information on total energy intake, medication use, clinical diagnoses, socioeconomic indicators, detailed lipid fractions, and other potentially relevant confounders. A prespecified country-specific EQ-5D value set should also be applied when calculation of a utility index is intended.

The cross-sectional design is central to the interpretation of all associations observed in MEDINAV. For anthropometric outcomes, the observed relationships with BMI and waist circumference cannot establish whether greater Mediterranean diet adherence preceded lower adiposity or whether participants with higher body weight had previously modified their dietary habits in response to clinical advice or health concerns. This possibility is particularly relevant when interpreting the observed non-linear patterns.

For cardiometabolic outcomes, the inverse associations with blood pressure and fasting blood glucose represent adjusted differences measured at a single point in time and cannot establish that greater Mediterranean diet adherence produced subsequent reductions in these parameters. Reverse causation is possible because participants with hypertension, impaired glucose regulation, dyslipidaemia, or other cardiovascular risk factors may have changed their diet before study participation. Furthermore, information on these diagnoses and their pharmacological treatment was unavailable for adjustment.

Similar caution applies to the psychological and health-related quality-of-life findings. Fewer anxiety symptoms or better self-perceived health may facilitate healthier dietary behaviours, while dietary habits may also be associated with psychological well-being through biological or behavioural pathways. The direction of these relationships cannot be determined from the present data. The absence of an association with depressive symptoms should likewise not be interpreted as evidence that Mediterranean diet adherence has no prospective relationship with depression.

Consequently, the associations reported in MEDINAV should be interpreted as cross-sectional relationships rather than evidence of causal effects, preventive effects, or therapeutic effects. Prospective studies with repeated dietary and outcome measurements, and intervention studies, where appropriate, are required to establish temporal sequence and evaluate whether changes in Mediterranean diet adherence precede changes in the outcomes examined.

This study has several strengths. It included 495 adults recruited from primary care centres under routine clinical-care conditions, thereby reflecting a real-world primary care setting. Complete data were available for the variables included in the principal regression analyses and for the individual EQ-5D-3L dimensions. The validated MEDAS-14 was used, and adherence was examined at both the total-score and individual-component levels [8,9]. The scoring criteria and operational definitions of all 14 MEDAS-14 components were transparently documented, and the sum of the component scores reproduced the recorded total score for all 495 participants. The study simultaneously evaluated anthropometric, clinical, psychological, and health-related quality-of-life outcomes. The robustness of the findings was examined through progressive covariate adjustment, HC3 heteroscedasticity-consistent standard errors, exclusion of the wine component, additional adjustment for current smoking or for any versus no alcohol consumption in separate sensitivity models, categorical modelling, assessment of non-linearity, false-discovery-rate correction across the nine principal outcome models, and sex-interaction analyses with corresponding multiplicity correction.

Several limitations should be acknowledged. First, the cross-sectional design precludes establishing temporality or causality and is susceptible to reverse causation. Participants with obesity, hypertension, diabetes, or other health concerns may have modified their dietary habits before study participation. Second, although recruitment under routine primary care conditions enhances the real-world relevance of the study, participants were recruited from a limited number of primary care centres within a single region. This may limit the generalisability of the findings and may also introduce selection bias.

Third, dietary adherence, physical activity, smoking, and alcohol consumption were self-reported and may therefore have been affected by recall and social-desirability bias. Although the MEDAS-14 is a validated instrument, it remains a brief dietary screener and does not provide detailed information on total energy intake, nutrient intake, or the complete dietary pattern. Furthermore, the low-, moderate-, and high-adherence categories used in the secondary analyses were exploratory and do not represent independently validated clinical thresholds. Alcohol intake could not be quantified precisely from the available response categories for drinks consumed on a typical drinking day. Residual confounding by alcohol consumption therefore cannot be excluded, although the principal associations were materially unchanged in a sensitivity analysis that additionally adjusted for any versus no alcohol consumption.

Fourth, residual or unmeasured confounding by medication use, comorbidities, socioeconomic circumstances, occupation, total energy intake, and other lifestyle or psychosocial factors cannot be excluded. In particular, information on diabetes status, glucose-lowering medication use, antihypertensive treatment, and lipid-lowering medication use was not included in the adjustment set, which limits the interpretation of the corresponding cardiometabolic associations. Fasting insulin concentrations and direct measures of insulin resistance, such as HOMA-IR, were also unavailable; therefore, insulin resistance could not be evaluated as a potential mediator of the observed associations. Although the principal models adjusted for age, sex, educational attainment, physical activity, and BMI when appropriate, these covariates cannot account for all potential sources of confounding.

Fifth, several outcomes and secondary analyses were examined, increasing the possibility of chance findings due to multiple testing. Although the principal analyses emphasised effect estimates and 95% confidence intervals, the same exposure was evaluated across nine outcomes. Benjamini–Hochberg false-discovery-rate correction was therefore additionally applied across the nine principal outcome models to address multiplicity and aid interpretation of the overall pattern of findings. The principal associations with BMI, systolic blood pressure, diastolic blood pressure, fasting blood glucose, Goldberg anxiety score, and EQ VAS remained statistically evident after FDR correction, whereas waist circumference, total cholesterol, and Goldberg depression score did not. The categorical and non-linear secondary analyses remained exploratory and were not comprehensively adjusted for multiple comparisons. False-discovery-rate correction was also applied across the nine formal sex-interaction tests. Findings from individual outcomes and secondary analyses, particularly the non-linear and categorical results for total cholesterol and the exploratory sex interactions, should therefore continue to be interpreted cautiously and warrant independent replication.

Finally, the quadratic analyses were based on fewer observations toward the extremes of the observed MEDAS-14 score distribution, where the corresponding confidence intervals were wider. The fitted U-shaped patterns for BMI, waist circumference, and total cholesterol may therefore partly reflect data sparsity at the extremes or the functional form imposed by the quadratic models. These curves should not be interpreted as demonstrating that very high Mediterranean diet adherence is harmful or that a MEDAS-14 score of approximately 9–10 represents an optimal biological threshold. Confirmation in larger samples using more flexible modelling approaches would be warranted.

## 5. Conclusions

In this cross-sectional study of adults attending primary care centres in Navarra, higher MEDAS-14 scores were associated, after adjustment, with lower systolic and diastolic blood pressure, lower fasting blood glucose, fewer anxiety symptoms, and better self-perceived health. These associations remained generally consistent in sensitivity analyses using HC3 heteroscedasticity-consistent standard errors, a 13-item adherence score excluding the wine component, and additional adjustment for current smoking or for any versus no alcohol consumption. The principal associations with BMI, systolic blood pressure, diastolic blood pressure, fasting blood glucose, anxiety symptoms, and EQ VAS remained statistically evident after Benjamini–Hochberg false-discovery-rate correction across the nine principal outcome models.

The principal linear model also showed an inverse association with BMI, whereas the association with waist circumference was not statistically significant after full adjustment; both anthropometric associations were attenuated after additional adjustment for current smoking. BMI, waist circumference, and total cholesterol showed evidence of non-linearity, and no statistically significant adjusted linear association was observed for total cholesterol. These findings should therefore not be interpreted as constant linear dose–response relationships across the entire MEDAS-14 score range. There was no evidence of an adjusted association between the MEDAS-14 score and depressive symptoms.

Given the cross-sectional design, the observed associations cannot establish temporality or causality. Interpretation of the cardiometabolic associations is additionally limited by the absence of information on relevant clinical diagnoses and medication use. The low-, moderate-, and high-adherence categories were used for exploratory analyses and should not be interpreted as independently validated clinical thresholds. Prospective and intervention studies are needed to determine whether changes in Mediterranean diet adherence precede subsequent changes in cardiometabolic health, psychological well-being, and health-related quality of life, and to further investigate the non-linear patterns observed in the present study.

## Figures and Tables

**Figure 1:**
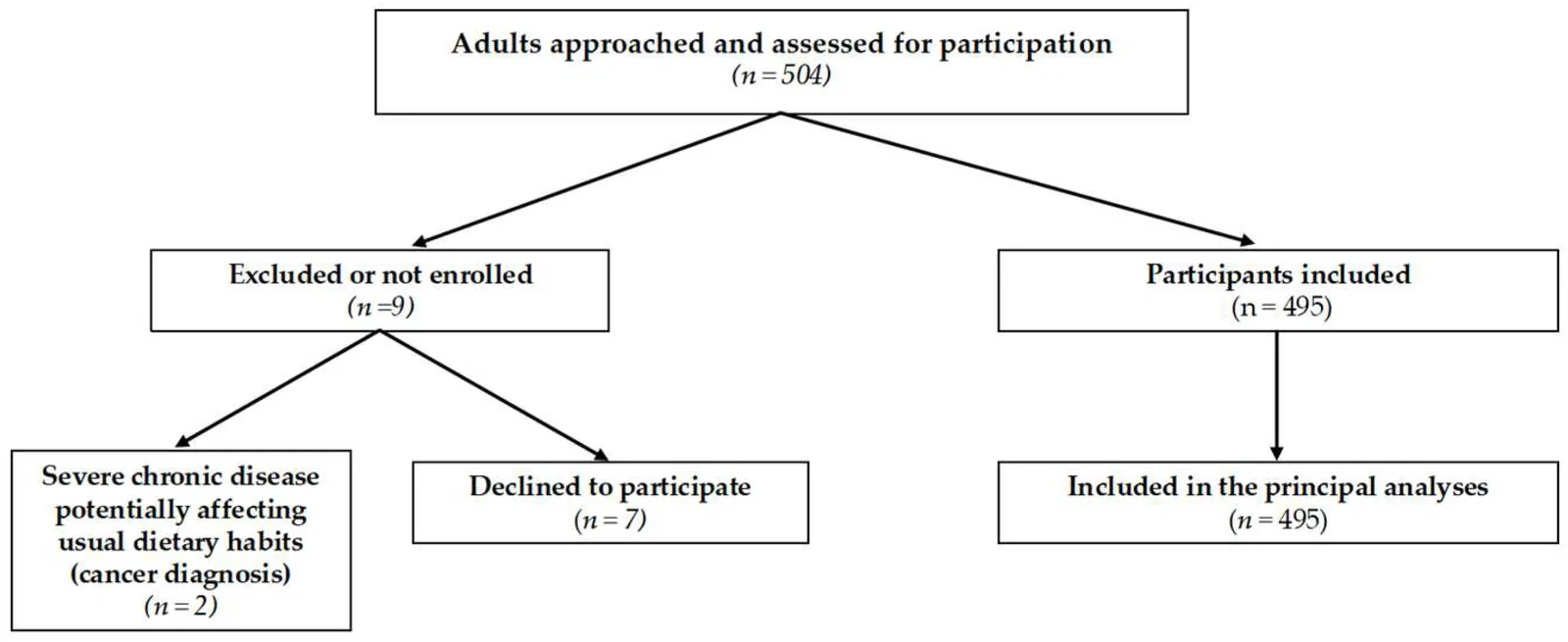
Flow diagram of participant recruitment and inclusion in the MEDINAV study. A total of 504 adults were approached and assessed for participation. Two were excluded because their cancer diagnoses met the criterion of severe chronic disease potentially affecting usual dietary habits, and seven declined to participate. The final analytical sample comprised 495 participants.

**Table 1:** Characteristics of the study population overall and by sex (*n* = 495).

Characteristic	Total (*n* = 495)	Men (*n* = 205)	Women (*n* = 290)	*p* Value
Sociodemographic characteristics				
Age, years	48.3 ± 15.0	51.3 ± 15.0	46.2 ± 14.6	<0.001
Educational attainment, *n* (%)				<0.001
Basic	46 (9.3)	23 (11.2)	23 (7.9)	
Secondary or vocational	192 (38.8)	96 (46.8)	96 (33.1)	
University or higher	257 (51.9)	86 (42.0)	171 (59.0)	
Lifestyle characteristics				
Physical activity, *n* (%)				<0.001
Low or inactive	91 (18.4)	28 (13.7)	63 (21.7)	
Moderate	137 (27.7)	96 (46.8)	41 (14.1)	
High	267 (53.9)	81 (39.5)	186 (64.1)	
Cigarette consumption, *n* (%)				<0.001 ^a^
None	438 (88.5)	171 (83.4)	267 (92.1)	
Fewer than 10 cigarettes/day	42 (8.5)	29 (14.1)	13 (4.5)	
11–20 cigarettes/day	9 (1.8)	1 (0.5)	8 (2.8)	
21–30 cigarettes/day	2 (0.4)	1 (0.5)	1 (0.3)	
More than 30 cigarettes/day	4 (0.8)	3 (1.5)	1 (0.3)	
Alcohol-consumption frequency, *n* (%)				<0.001 ^a^
Never	98 (19.8)	19 (9.3)	79 (27.2)	
Once per month or less	192 (38.8)	86 (42.0)	106 (36.6)	
2–4 times per month	169 (34.1)	76 (37.1)	93 (32.1)	
2–4 times per week	32 (6.5)	21 (10.2)	11 (3.8)	
≥4 times per week	4 (0.8)	3 (1.5)	1 (0.3)	
Anthropometric characteristics				
Body mass index, kg/m^2^	25.6 ± 4.9	26.2 ± 4.3	25.2 ± 5.3	0.021
Waist circumference, cm	88.5 ± 12.9	92.5 ± 10.9	85.6 ± 13.4	<0.001
BMI category, *n* (%)				0.021 ^a^
Underweight	8 (1.6)	0 (0.0)	8 (2.8)	
Normal weight	258 (52.1)	100 (48.8)	158 (54.5)	
Overweight	126 (25.5)	62 (30.2)	64 (22.1)	
Obesity	103 (20.8)	43 (21.0)	60 (20.7)	
Clinical characteristics				
Systolic blood pressure, mmHg	125.1 ± 15.7	128.6 ± 13.8	122.7 ± 16.6	<0.001
Diastolic blood pressure, mmHg	77.7 ± 9.6	80.8 ± 9.6	75.6 ± 9.0	<0.001
Fasting blood glucose, mg/dL	86.2 ± 14.8	88.6 ± 14.7	84.4 ± 14.5	0.002
Total cholesterol, mg/dL	197.4 ± 39.6	194.7 ± 38.2	199.3 ± 40.4	0.196
Mediterranean diet adherence				
MEDAS-14 score	8.82 ± 2.03	8.05 ± 1.79	9.36 ± 2.02	<0.001
Exploratory adherence categories, *n* (%)				<0.001
Low, ≤7 points	130 (26.3)	85 (41.5)	45 (15.5)	
Moderate, 8–9 points	224 (45.3)	89 (43.4)	135 (46.6)	
High, ≥10 points	141 (28.5)	31 (15.1)	110 (37.9)	
Psychological symptoms and perceived health				
Goldberg anxiety score	3.70 ± 2.66	3.37 ± 2.55	3.93 ± 2.71	0.018
Goldberg depression score	2.35 ± 2.45	2.00 ± 2.47	2.60 ± 2.42	0.007
EuroQol VAS score	73.2 ± 14.3	73.7 ± 14.1	72.9 ± 14.4	0.540

Note: Data are presented as mean ± standard deviation or *n* (column percentage). Continuous variables were compared using Welch’s *t*-test. Categorical variables were compared using Pearson’s chi-squared test, except for BMI category, cigarette consumption, and alcohol-consumption frequency, which were evaluated using an exact test with Monte Carlo simulation because of sparse or zero-frequency cells. Each *p* value reported for a categorical block represents the global comparison across all categories. The adherence categories were used for exploratory analyses. All tests were two-sided. BMI: body mass index; MEDAS-14: 14-item Mediterranean Diet Adherence Screener; VAS: visual analogue scale. ^a^ Exact *p* value estimated using 200,000 Monte Carlo samples with fixed marginal totals.

**Table 2:** Unadjusted differences in sociodemographic, lifestyle, clinical, and psychological characteristics between women and men.

Variable	Effect Estimate	95% CI	*p* Value
Sociodemographic and lifestyle characteristics			
Age, years	β = −5.16	−7.82 to −2.49	<0.001
University education or higher	OR = 1.99	1.38 to 2.86	<0.001
High physical activity	OR = 2.74	1.89 to 3.96	<0.001
Current smoking	OR = 0.43	0.25 to 0.76	0.004 ^a^
Alcohol consumption ≥ 2 times/week	OR = 0.33	0.16 to 0.67	0.002 ^a^
Anthropometric characteristics			
Body mass index, kg/m^2^	β = −1.00	−1.85 to −0.15	0.021
Waist circumference, cm	β = −6.93	−9.08 to −4.78	<0.001
Obesity	OR = 0.98	0.63 to 1.53	0.938
Clinical characteristics			
Systolic blood pressure, mmHg	β = −5.95	−8.64 to −3.26	<0.001
Diastolic blood pressure, mmHg	β = −5.18	−6.86 to −3.50	<0.001
Fasting blood glucose, mg/dL	β = −4.19	−6.82 to −1.56	0.002
Total cholesterol, mg/dL	β = 4.63	−2.40 to 11.65	0.196
Mediterranean diet adherence			
MEDAS-14 score	β = 1.31	0.97 to 1.65	<0.001
Psychological symptoms and perceived health			
Goldberg anxiety score	β = 0.57	0.10 to 1.03	0.018
Goldberg depression score	β = 0.60	0.16 to 1.04	0.007
EuroQol VAS score	β = −0.80	−3.35 to 1.76	0.540

Note: For continuous variables, β represents the unadjusted mean difference calculated as women minus men; negative coefficients indicate lower mean values among women. For binary variables, OR represents the unadjusted odds ratio comparing women with men. Continuous variables were analysed using Welch’s *t*-test. Binary variables were analysed using Pearson’s chi-squared test, except for current smoking and alcohol consumption ≥ 2 times/week, for which Fisher’s exact test was used. All tests were two-sided. CI: confidence interval; MEDAS-14: 14-item Mediterranean Diet Adherence Screener; OR: odds ratio; VAS: visual analogue scale. ^a^ Fisher’s exact test.

**Table 3:** Distribution of the 14 Mediterranean Diet Adherence Screener (MEDAS-14) components (*n* = 495).

No.	MEDAS-14 Component	Criterion for 1 Point	*n*	%
1	Olive oil as the principal culinary fat	Yes	466	94.1
2	Total olive oil consumption	≥4 tablespoons/day	409	82.6
3	Vegetables or greens	≥2 servings/day, including ≥1 serving raw or as salad	327	66.1
4	Fruit, including natural fruit juice	≥3 servings/day	221	44.6
5	Red or processed meat	<1 serving/day	432	87.3
6	Butter, margarine, or cream	<1 serving/day	410	82.8
7	Sugar-sweetened or carbonated beverages	<1 beverage/day	403	81.4
8	Wine consumption	≥7 glasses/week	25	5.1
9	Legumes	≥3 servings/week	161	32.5
10	Fish or seafood	≥3 servings/week	171	34.5
11	Commercial pastries, biscuits, or sweets	<2 servings/week	344	69.5
12	Nuts	≥3 servings/week	305	61.6
13	Preference for poultry or rabbit over red or processed meat	Yes	369	74.5
14	Sofrito prepared with tomato, garlic, onion or leek, and olive oil	≥2 times/week	322	65.1

Note: Values are presented as the number and percentage of participants who met the corresponding validated MEDAS-14 criterion and received one point for that component. Each component was scored as 0 or 1, producing a total score ranging from 0 to 14, with higher scores indicating greater adherence to the Mediterranean diet. No component data were missing. The sum of the 14 component scores reproduced the recorded MEDAS-14 total score for all 495 participants. MEDAS-14: 14-item Mediterranean Diet Adherence Screener.

**Table 4:** MEDAS-14 score according to sociodemographic, lifestyle, and anthropometric characteristics.

Characteristic	*n*	MEDAS-14 Score, Mean ± SD	*p* Value
Sex			<0.001 ^a^
Men	205	8.05 ± 1.79	
Women	290	9.36 ± 2.02	
Age group, years			0.008 ^b^
18–39	159	8.45 ± 2.13	
40–59	212	8.87 ± 1.96	
≥60	124	9.20 ± 1.95	
Educational attainment			<0.001 ^b^
Basic	46	9.33 ± 2.27	
Secondary or vocational	192	8.29 ± 2.03	
University or higher	257	9.12 ± 1.91	
Physical activity			<0.001 ^b^
Low or inactive	91	8.47 ± 2.24	
Moderate	137	7.81 ± 1.69	
High	267	9.45 ± 1.88	
BMI category			<0.001 ^c^
Underweight	8	9.25 ± 0.71	
Normal weight	258	9.22 ± 1.72	
Overweight	126	8.60 ± 2.26	
Obesity	103	8.04 ± 2.27	
Cigarette consumption			<0.001 ^c^
None	438	9.08 ± 1.95	
Fewer than 10 cigarettes/day	42	6.90 ± 1.10	
11–20 cigarettes/day	9	7.22 ± 2.22	
21–30 cigarettes/day	2	5.00 ± 1.41	
More than 30 cigarettes/day	4	5.50 ± 1.00	
Alcohol-consumption frequency			<0.001 ^c^
Never	98	9.40 ± 1.90	
Once per month or less	192	8.95 ± 1.86	
2–4 times per month	169	8.32 ± 2.09	
2–4 times per week	32	9.16 ± 2.49	
≥4 times per week	4	6.50 ± 1.00	

Note: Values are presented as mean ± standard deviation. The *p* values represent global comparisons within each characteristic and should not be interpreted as tests of linear trend. All tests were two-sided. BMI: body mass index; MEDAS-14: 14-item Mediterranean Diet Adherence Screener; SD: standard deviation. ^a^ Welch’s *t*-test. ^b^ Welch’s one-way analysis of variance. ^c^ Kruskal–Wallis test; categories without observations were excluded from the corresponding.

## Data Availability

The data supporting the findings of this study are available from the corresponding author upon reasonable request. The data are not publicly available because they contain potentially identifiable clinical and health-related information and are subject to ethical and data-protection restrictions.

## Supplementary Materials

The following supporting information can be downloaded at https://www.mdpi.com/article/10.3390/nu18193156/s1. Figure S1: Adjusted non-linear associations between the MEDAS-14 score and body mass index, waist circumference, and total cholesterol; Table S1: Coding and operational definitions of the variables included in the analyses; Table S2: Progressive regression models of the associations between the MEDAS-14 score and study outcomes; Table S3: Sensitivity analyses of the associations between the MEDAS-14 score and study outcomes; Table S4: Sex-specific associations between the MEDAS-14 score and study outcomes and interaction tests; Table S5: Distribution of the individual EQ-5D-3L dimensions; Table S6: Distribution of lifestyle habits and family cardiovascular history.

## Abbreviations

The following abbreviations are used in this manuscript:

BMI	Body mass index
CI	Confidence interval
EQ-5D-3L	EuroQol five-dimension, three-level questionnaire
EQ VAS	EuroQol visual analogue scale
FDR	False discovery rate
HC3	Heteroscedasticity-consistent type 3
IPAQ	International Physical Activity Questionnaire
MEDAS-14	14-item Mediterranean Diet Adherence Screener
MEDINAV	Mediterranean Diet Study in the Adult Population of Navarra
OR	Odds ratio
PREDIMED	Prevención con Dieta Mediterránea
SD	Standard deviation
SF-12	12-item Short Form Health Survey
SF-36	36-item Short Form Health Survey
STROBE	Strengthening the Reporting of Observational Studies in Epidemiology
STROBE-nut	Strengthening the Reporting of Observational Studies in Epidemiology—Nutritional Epidemiology
SUN	University of Navarra Follow-Up Study
