# Supplementary material for: Associations of Mediterranean Diet Adherence with Cardiometabolic Outcomes, Psychological Symptoms, and Health-Related Quality of Life Among Adults Attending Primary Care Centres in Navarra, Spain: A Cross-Sectional Study
Source: Nutrients. 2026 Sep 25;18(19):3156. doi: 10.3390/nu18193156 (PMC13666617; doi:10.3390/nu18193156)
Supplement: Supplementary file 1 [file nutrients-18-03156-s001.zip › nutrients-4532025-supplementary.pdf]

Supplementary Table S1. Coding and operational definitions of the variables included in the analyses

Domain	Database variable	Analytical variable	Coding or operational definition	Role in analyses
**Identification**	ID	Original participant identifier	Unique original numeric participant code; 495 unique identifiers in the verified database	Identification only
**Eligibility and data availability**	CONSENTIMIENTO	Informed consent	SI = written informed consent available	Eligibility
Eligibility and data availability	ANALITICA	Laboratory data available	SI = laboratory measurements available	Data availability
**Sociodemographic characteristics**	EDAD	Age	Continuous, years	Covariate
Sociodemographic characteristics	EDAD	Age group	18–39, 40–59, or ≥60 years	Descriptive/exploratory
Sociodemographic characteristics	SEXO	Sex	M = men; F = women	Covariate/effect modifier
Sociodemographic characteristics	NIVEL ESTUDIOS	Basic education	Primary education, school graduate, or compulsory secondary education	Covariate
Sociodemographic characteristics	NIVEL ESTUDIOS	Secondary or vocational education	Upper secondary, intermediate vocational, or advanced vocational education	Covariate
Sociodemographic characteristics	NIVEL ESTUDIOS	University education or higher	Diploma, university degree, licenciatura, master's degree, or doctorate	Covariate
Sociodemographic characteristics	OCUPACION	Occupation	Original questionnaire categories; not included in the principal models	Descriptive/potential covariate
**Tobacco use**	HABITOS1	Cigarette consumption	A = none; B = <10/day; C = 11–20/day; D = 21–30/day; E = >30/day	Descriptive
Tobacco use	HABITOS1	Current smoking	No = category A; yes = categories B–E	Sensitivity covariate
**Alcohol use**	HABITOS3	Alcohol-consumption frequency	A = never; B = once/month or less; C = 2–4 times/month; D = 2–4 times/week; E = ≥4 times/week	Descriptive
Alcohol use	HABITOS3	Any alcohol consumption	No = category A (never); Yes = categories B–E	Sensitivity covariate
Alcohol use	HABITOS4	Alcoholic drinks per drinking day	0 = not applicable because HABITOS3 = A (never; questionnaire skip); A = 1–2; B = 3–4; C = 5–6; D = 7–8; E = ≥10 drinks/day	Source/descriptive variable
Alcohol use	HABITOS3	Alcohol consumption ≥2 times/week	No = categories A–C; yes = categories D–E	Exploratory variable
**Physical activity**	ACTFISICA1–ACTFISICA7	IPAQ short-form responses	Frequency and duration of vigorous activity, moderate activity, walking, and sitting	Source variables
Physical activity	Act_vigorosa	Vigorous physical activity	Database-derived physical-activity value	Intermediate variable
Physical activity	Act_moderada	Moderate physical activity	Database-derived physical-activity value	Intermediate variable
Physical activity	Act_Caminata	Walking	Database-derived walking value	Intermediate variable
Physical activity	ACTFISICA_TOTAL	Total physical activity	Database-derived total physical-activity value	Intermediate variable
Physical activity	ACTFISICA_INTERPRETACION	Physical-activity category	Low/inactive, moderate, or high	Covariate
**Anthropometric characteristics**	PESO	Body weight	Continuous, kg	Source variable
Anthropometric characteristics	TALLA	Height	Continuous, m	Source variable
Anthropometric characteristics	IMC	Body mass index	Weight (kg) divided by height squared (m^2)	Outcome/covariate
Anthropometric characteristics	R_ IMC	BMI category	Underweight: <18.5; normal weight: 18.5–<25; overweight: 25–<30; obesity: ≥30 kg/m^2	Descriptive/exploratory

Domain	Database variable	Analytical variable	Coding or operational definition	Role in analyses
Anthropometric characteristics	CINTURA	Waist circumference	Continuous, cm	Outcome
Anthropometric characteristics	CADERA	Hip circumference	Continuous, cm	Supplementary variable
Anthropometric characteristics	I_CC	Waist-to-hip ratio	Waist circumference divided by hip circumference	Supplementary variable
Anthropometric characteristics	R_ICC	Waist-to-hip risk category	Database-derived category	Supplementary variable
**Blood pressure**	TensiónArterial1	Systolic blood pressure	Continuous, mmHg; mean of the second and third measurements	Outcome
Blood pressure	TensiónArterial2	Diastolic blood pressure	Continuous, mmHg; mean of the second and third measurements	Outcome
**Biochemical measurements**	glucosa(mg/dl)	Fasting blood glucose	Continuous, mg/dL	Outcome
Biochemical measurements	I_ GLUCOSA	Fasting-glucose category	Database-derived clinical classification; not used in the principal regression models	Supplementary variable
Biochemical measurements	Colesterol(mg/dl)	Total cholesterol	Continuous, mg/dL	Outcome
Biochemical measurements	I_Colesterol	Total-cholesterol category	Database-derived clinical classification; not used in the principal regression models	Supplementary variable
**Mediterranean diet adherence**	1_DM1	Olive oil as principal culinary fat	1 = yes; 0 = no	Exposure component
Mediterranean diet adherence	2_DM2	Total olive oil consumption	1 = ≥4 tablespoons/day; 0 = below criterion	Exposure component
Mediterranean diet adherence	3_DM3	Vegetables or greens	1 = ≥2 servings/day, including ≥1 serving raw or as salad; 0 = below criterion	Exposure component
Mediterranean diet adherence	4_DM4	Fruit, including natural fruit juice	1 = ≥3 servings/day; 0 = below criterion	Exposure component
Mediterranean diet adherence	5_DM5	Red or processed meat	1 = <1 serving/day; 0 = criterion not met	Exposure component
Mediterranean diet adherence	6_DM6	Butter, margarine, or cream	1 = <1 serving/day; 0 = criterion not met	Exposure component
Mediterranean diet adherence	7_DM7	Sugar-sweetened or carbonated beverages	1 = <1 beverage/day; 0 = criterion not met	Exposure component
Mediterranean diet adherence	8_DM8	Wine consumption	1 = ≥7 glasses/week; 0 = below criterion	Exposure component
Mediterranean diet adherence	9_DM9	Legumes	1 = ≥3 servings/week; 0 = below criterion	Exposure component
Mediterranean diet adherence	10_DM10	Fish or seafood	1 = ≥3 servings/week; 0 = below criterion	Exposure component
Mediterranean diet adherence	11_DM11	Commercial pastries, biscuits, or sweets	1 = <2 servings/week; 0 = criterion not met	Exposure component
Mediterranean diet adherence	12_DM12	Nuts	1 = ≥3 servings/week; 0 = below criterion	Exposure component
Mediterranean diet adherence	13_DM13	Preference for poultry or rabbit over red or processed meat	1 = yes; 0 = no	Exposure component
Mediterranean diet adherence	14_DM14	Sofrito prepared with tomato, garlic, onion or leek, and olive oil	1 = ≥2 times/week; 0 = below criterion	Exposure component
Mediterranean diet adherence	DM_TOTAL	MEDAS-14 score	Sum of 1_DM1–14_DM14; theoretical range 0–14 and observed range 4–14, with higher scores indicating greater adherence. The calculated sum reproduced the recorded total for all 495 participants.	Primary exposure

Domain	Database variable	Analytical variable	Coding or operational definition	Role in analyses
Mediterranean diet adherence	DM_TOTAL	Low adherence	≤7 points	Exploratory exposure category
Mediterranean diet adherence	DM_TOTAL	Moderate adherence	8–9 points	Exploratory exposure category
Mediterranean diet adherence	DM_TOTAL	High adherence	≥10 points	Exploratory exposure category
Mediterranean diet adherence	DM_TOTAL − 8_DM8	MEDAS-14 score excluding wine	Sum of the 13 MEDAS-14 components other than wine; theoretical range 0–13 and observed range 4–13. Used only to assess whether the principal associations depended on the component awarding one point for wine consumption	Sensitivity exposure
Mediterranean diet adherence	Derived	Mean-centred MEDAS-14 score	MEDAS-14 score minus its sample mean	Non-linearity analysis
Mediterranean diet adherence	Derived	Quadratic MEDAS-14 term	Square of the mean-centred MEDAS-14 score	Non-linearity analysis
**Goldberg anxiety symptoms**	EADG_ANSIEDAD1–EADG_ANSIEDAD9	Individual anxiety items	No = 0; yes = 1	Source variables
Goldberg anxiety symptoms	TOTAL_ANSIEDAD	Goldberg anxiety score	Sum of nine anxiety items; range 0–9, with higher scores indicating more symptoms	Outcome
**Goldberg depressive symptoms**	EADG_DEPRESION1–EADG_DEPRESION9	Individual depression items	No = 0; yes = 1	Source variables
Goldberg depressive symptoms	TOTAL_DEPRESION	Goldberg depression score	Sum of nine depression items; range 0–9, with higher scores indicating more symptoms	Outcome
**EQ-5D-3L dimensions**	EUROQOL1	Mobility	Standardized Spanish response labels, analytically recoded as 1 = no problems; 2 = some problems; 3 = confined to bed	Supplementary outcome
EQ-5D-3L dimensions	EUROQOL2	Self-care	Standardized Spanish response labels, analytically recoded as 1 = no problems; 2 = some problems; 3 = unable to wash or dress	Supplementary outcome
EQ-5D-3L dimensions	EUROQOL3	Usual activities	Standardized Spanish response labels, analytically recoded as 1 = no problems; 2 = some problems; 3 = unable to perform usual activities	Supplementary outcome
EQ-5D-3L dimensions	EUROQOL4	Pain/discomfort	Standardized Spanish response labels, analytically recoded as 1 = no pain/discomfort; 2 = moderate pain/discomfort; 3 = extreme pain/discomfort	Supplementary outcome
EQ-5D-3L dimensions	EUROQOL5	Anxiety/depression	Standardized Spanish response labels, analytically recoded as 1 = not anxious/depressed; 2 = moderately anxious/depressed; 3 = extremely anxious/depressed	Supplementary outcome
**Perceived health**	EUROQOL6	EuroQol VAS score	Continuous, 0–100; higher scores indicate better self-perceived health	Outcome
**Interaction terms**	Derived	MEDAS-14 score × sex	Product of the continuous MEDAS-14 score and the female-sex indicator	Exploratory effect-modification analysis

Note: The primary MEDAS-14 score included all 14 components, including the wine-consumption component. A 13-item score excluding wine was used only as a sensitivity analysis to assess whether the principal associations depended on the component awarding one point for wine consumption. In the updated questionnaire, a MEDAS criterion was marked only when it awarded one point; unmarked criteria were coded as 0 in the database. For alcohol-use question 4, participants reporting never drinking in question 3 skipped the question and were coded 0 (not applicable) in HABITOS4. A binary indicator of any versus no alcohol consumption was derived from HABITOS3, with category A (never) classified as no alcohol consumption and categories B–E classified as any alcohol consumption, and was used only as a sensitivity covariate. The low-, moderate-, and high-adherence categories were used only in exploratory analyses. Educational attainment and physical activity were entered into the multivariable models as categorical variables. Models for BMI and waist circumference were adjusted for age, sex, educational attainment, and physical activity. Models for blood pressure, fasting blood glucose, total cholesterol, Goldberg anxiety and depression scores, and EuroQol VAS were additionally adjusted for BMI. BMI: body mass index; EQ-5D-3L: EuroQol five-dimension, three-level questionnaire; IPAQ: International Physical Activity Questionnaire; MEDAS-14: 14-item Mediterranean Diet Adherence Screener; VAS: visual analogue scale.

Supplementary Table S2. Progressive regression models of the associations between the MEDAS-14 score and study outcomes

Outcome	Crude β (95% CI)	*p* value	Age- and sex-adjusted β (95% CI)	*p* value	Fully adjusted β (95% CI)	*p* value
**Anthropometric outcomes**						
Body mass index, kg/m²	−0.575 (−0.784 to −0.367)	<0.001	−0.625 (−0.848 to −0.402)	<0.001	−0.299 (−0.521 to −0.077)a	0.009
Waist circumference, cm	−1.288 (−1.837 to −0.738)	<0.001	−1.184 (−1.741 to −0.626)	<0.001	−0.475 (−1.046 to 0.095)a	0.102
**Clinical outcomes**						
Systolic blood pressure, mmHg	−0.931 (−1.611 to −0.250)	0.007	−1.154 (−1.822 to −0.486)	<0.001	−1.056 (−1.774 to −0.338)b	0.004
Diastolic blood pressure, mmHg	−0.880 (−1.291 to −0.470)	<0.001	−0.836 (−1.246 to −0.426)	<0.001	−0.838 (−1.261 to −0.415)b	<0.001
Fasting blood glucose, mg/dL	−1.238 (−1.871 to −0.606)	<0.001	−1.492 (−2.138 to −0.847)	<0.001	−0.745 (−1.365 to −0.125)b	0.019
Total cholesterol, mg/dL	0.257 (−1.466 to 1.979)	0.770	−1.525 (−3.269 to 0.220)	0.087	−0.823 (−2.621 to 0.975)b	0.369
**Psychological outcomes**						
Goldberg anxiety score	−0.284 (−0.397 to −0.171)	<0.001	−0.316 (−0.433 to −0.198)	<0.001	−0.249 (−0.368 to −0.130)b	<0.001
Goldberg depression score	−0.011 (−0.118 to 0.096)	0.842	−0.049 (−0.163 to 0.065)	0.398	0.033 (−0.086 to 0.152)b	0.590
**Health-related quality of life**						
EQ VAS score	1.240 (0.629 to 1.852)	<0.001	1.606 (0.953 to 2.260)	<0.001	1.224 (0.570 to 1.878)b	<0.001

Note: Regression coefficients represent the mean difference in each outcome associated with a one-point increase in the MEDAS-14 score. Separate ordinary least-squares regression models were estimated for each outcome. The crude model included only the continuous MEDAS-14 score. The age- and sex-adjusted model included the continuous MEDAS-14 score, age, and sex. Evidence of non-linearity for body mass index, waist circumference, and total cholesterol is examined in Supplementary Table S3 and Supplementary Figure S1. All tests were two-sided. BMI: body mass index; CI: confidence interval; EQ VAS: EuroQol visual analogue scale; MEDAS-14: 14-item Mediterranean Diet Adherence Screener.

[a] Fully adjusted for age, sex, educational attainment, and physical activity. BMI was not included in the BMI or waist-circumference models.

[b] Fully adjusted for age, sex, educational attainment, physical activity, and BMI. Educational attainment and physical activity were entered as categorical variables.

Supplementary Table S3. Sensitivity analyses of the associations between the MEDAS-14 score and study outcomes

Panel A. Robustness and non-linearity analyses

Outcome	HC3 robust SE, β (95% CI); *p* value	MEDAS-14 score excluding wine, β (95% CI); *p* value	Additional adjustment for current smoking, β (95% CI); *p* value	Additional adjustment for any alcohol consumption, β (95% CI); p value	*p value for quadratic term*
**Anthropometric outcomes**					
Body mass index, kg/m²	−0.299 (−0.535 to −0.064); 0.013	−0.315 (−0.538 to −0.092); 0.006	−0.169 (−0.395 to 0.058); 0.144	−0.292 (−0.515 to −0.070); 0.010	<0.001
Waist circumference, cm	−0.475 (−0.960 to 0.009); 0.054	−0.469 (−1.042 to 0.105); 0.109	−0.338 (−0.929 to 0.252); 0.260	−0.507 (−1.074 to 0.060); 0.080	0.002
**Clinical outcomes**					
Systolic blood pressure, mmHg	−1.056 (−1.732 to −0.380); 0.002	−1.220 (−1.940 to −0.501); <0.001	−0.840 (−1.576 to −0.103); 0.026	−1.079 (−1.795 to −0.363); 0.003	0.745
Diastolic blood pressure, mmHg	−0.838 (−1.271 to −0.405); <0.001	−0.871 (−1.295 to −0.446); <0.001	−0.870 (−1.306 to −0.433); <0.001	−0.836 (−1.259 to −0.412); <0.001	0.206
Fasting blood glucose, mg/dL	−0.745 (−1.233 to −0.258); 0.003	−0.649 (−1.273 to −0.025); 0.042	−0.640 (−1.279 to −0.002); 0.049	−0.748 (−1.369 to −0.127); 0.018	0.483
Total cholesterol, mg/dL	−0.823 (−2.906 to 1.260); 0.438	−1.298 (−3.102 to 0.507); 0.158	−1.507 (−3.345 to 0.330); 0.108	−0.787 (−2.584 to 1.010); 0.390	<0.001
**Psychological outcomes**					
Goldberg anxiety score	−0.249 (−0.377 to −0.121); <0.001	−0.214 (−0.334 to −0.093); <0.001	−0.241 (−0.364 to −0.118); <0.001	−0.254 (−0.373 to −0.136); <0.001	0.604
Goldberg depression score	0.033 (−0.086 to 0.151); 0.587	0.064 (−0.055 to 0.184); 0.291	0.046 (−0.076 to 0.169); 0.458	0.030 (−0.089 to 0.149); 0.619	0.336
**Health-related quality of life**					
EuroQol VAS score	1.224 (0.595 to 1.852); <0.001	1.188 (0.530 to 1.846); <0.001	1.136 (0.462 to 1.810); <0.001	1.228 (0.573 to 1.883); <0.001	0.252

Panel B. False-discovery-rate correction across the nine principal outcome models

Outcome	Principal-model *p* value	FDR-adjusted *q* value
Body mass index, kg/m²	0.009	0.015
Waist circumference, cm	0.102	0.132
Systolic blood pressure, mmHg	0.004	0.009
Diastolic blood pressure, mmHg	<0.001	<0.001
Fasting blood glucose, mg/dL	0.019	0.028
Total cholesterol, mg/dL	0.369	0.415
Goldberg anxiety score	<0.001	<0.001
Goldberg depression score	0.590	0.590
EuroQol VAS score	<0.001	<0.001

Panel C. Adjusted categorical associations for outcomes showing evidence of non-linearity

Outcome	Moderate versus low adherence, β (95% CI)	*p* value	High versus low adherence, β (95% CI)	*p* value	Global *p* value
Body mass index, kg/m²[a]	−2.949 (−3.980 to −1.917)	<0.001	−2.510 (−3.730 to −1.290)	<0.001	<0.001
Waist circumference, cm[a]	−5.461 (−8.137 to −2.785)	<0.001	−4.992 (−8.157 to −1.826)	0.002	<0.001
Total cholesterol, mg/dL[b]	−19.316 (−27.874 to −10.759)	<0.001	−7.901 (−17.872 to 2.070)	0.120	<0.001

Note: Panel A presents adjusted regression coefficients with 95% confidence intervals. The HC3 analysis used heteroscedasticity-consistent standard errors while retaining the exposure and covariates from the corresponding fully adjusted model. The score excluding wine was calculated by subtracting MEDAS-14 item 8 from the complete 14-item score, producing a 13-item score ranging from 0 to 13. This analysis was conducted only to assess whether the principal associations depended on the component awarding one point for wine consumption. The smoking-adjusted analysis added current smoking status to the fully adjusted model. The alcohol-adjusted analysis added a binary indicator of any versus no alcohol consumption to the fully adjusted model. Non-linearity was assessed by including a quadratic term for the mean-centred MEDAS-14 score.

Panel B presents Benjamini–Hochberg false-discovery-rate-adjusted q values calculated across the nine principal outcome models. Panel C presents adjusted mean differences using low adherence (≤7 points) as the reference category. Moderate adherence was defined as 8–9 points and high adherence as ≥10 points. These categories were used for exploratory analyses. Global p values test the joint contribution of the moderate- and high-adherence indicator variables. All tests were two-sided.

[a] Adjusted for age, sex, educational attainment, and physical activity.

[b] Adjusted for age, sex, educational attainment, physical activity, and body mass index.

CI: confidence interval; FDR: false discovery rate; HC3: heteroscedasticity-consistent covariance estimator; MEDAS-14: 14-item Mediterranean Diet Adherence Screener; SE: standard error; VAS: visual analogue scale.

Supplementary Table S4. Sex-specific associations between the MEDAS-14 score and study outcomes and interaction tests

Outcome	Men, adjusted β (95% CI); *p* value	Women, adjusted β (95% CI); *p* value	*p* value for MEDAS-14 score × sex interaction	FDR-adjusted *q* value
**Anthropometric outcomes**				
Body mass index, kg/m^2 [a]	−0.398 (−0.764 to −0.033); 0.033	−0.251 (−0.514 to 0.013); 0.062	0.501	0.564
Waist circumference, cm [a]	−1.257 (−2.192 to −0.323); 0.008	−0.095 (−0.769 to 0.578); 0.781	0.039	0.102
**Clinical outcomes**				
Systolic blood pressure, mmHg [b]	−1.417 (−2.594 to −0.240); 0.018	−0.882 (−1.729 to −0.035); 0.041	0.447	0.564
Diastolic blood pressure, mmHg [b]	−1.782 (−2.467 to −1.096); <0.001	−0.383 (−0.877 to 0.110); 0.128	<0.001	0.003
Fasting blood glucose, mg/dL [b]	0.053 (−0.960 to 1.066); 0.918	−1.130 (−1.859 to −0.400); 0.002	0.051	0.102
Total cholesterol, mg/dL [b]	−2.518 (−5.461 to 0.425); 0.093	−0.006 (−2.124 to 2.112); 0.996	0.154	0.231
**Psychological outcomes**				
Goldberg anxiety score [b]	−0.399 (−0.594 to −0.204); <0.001	−0.176 (−0.317 to −0.036); 0.014	0.056	0.102
Goldberg depression score [b]	0.048 (−0.147 to 0.244); 0.628	0.025 (−0.116 to 0.166); 0.725	0.844	0.844
**Health-related quality of life**				
EQ VAS score [b]	−0.267 (−1.327 to 0.793); 0.621	1.942 (1.179 to 2.705); <0.001	<0.001	0.003

Note: Sex-specific coefficients were derived from pooled multivariable models containing the continuous MEDAS-14 score, sex, and a MEDAS-14 score × sex interaction term. Regression coefficients represent the adjusted mean difference in each outcome associated with a one-point increase in the MEDAS-14 score within each sex. The interaction *p* value tests whether the association between the MEDAS-14 score and the corresponding outcome differs between women and men. Differences in the statistical significance of the sex-specific coefficients were not interpreted as evidence of effect modification unless supported by the formal interaction test. False-discovery-rate-adjusted *q* values were calculated across the nine interaction tests using the Benjamini–Hochberg procedure. These interaction analyses were considered exploratory. All tests were two-sided.

[a] Adjusted for age, educational attainment, and physical activity.

[b] Adjusted for age, educational attainment, physical activity, and body mass index.

CI: confidence interval; EQ VAS: EuroQol visual analogue scale; FDR: false discovery rate; MEDAS-14: 14-item Mediterranean Diet Adherence Screener.

Supplementary Table S5. Distribution of the individual EQ-5D-3L dimensions

EQ-5D-3L dimension and response level	n	%
**Mobility**		
Level 1: No problems walking	434	87.7
Level 2: Some problems walking	57	11.5
Level 3: Confined to bed	4	0.8
**Self-care**		
Level 1: No problems with self-care	469	94.7
Level 2: Some problems washing or dressing	24	4.8
Level 3: Unable to wash or dress	2	0.4
**Usual activities**		
Level 1: No problems performing usual activities	454	91.7
Level 2: Some problems performing usual activities	39	7.9
Level 3: Unable to perform usual activities	2	0.4
**Pain/discomfort**		
Level 1: No pain or discomfort	299	60.4
Level 2: Moderate pain or discomfort	154	31.1
Level 3: Extreme pain or discomfort	42	8.5
**Anxiety/depression**		
Level 1: Not anxious or depressed	229	46.3
Level 2: Moderately anxious or depressed	254	51.3
Level 3: Extremely anxious or depressed	12	2.4

Note: Values are presented as the number and percentage of the total study population (N = 495). Percentages were calculated using the total sample as the denominator. No data were missing for any EQ-5D-3L dimension. Spelling variants corresponding to the same response category in the original database were harmonised before analysis. EQ-5D-3L: EuroQol five-dimension, three-level questionnaire.

Supplementary Table S6. Distribution of lifestyle habits and family cardiovascular history

Questionnaire item and response category	Code	n	%
**HABITOS1. Average number of cigarettes smoked per day**			
None	A	438	88.5
Fewer than 10 cigarettes	B	42	8.5
11–20 cigarettes	C	9	1.8
21–30 cigarettes	D	2	0.4
More than 30 cigarettes	E	4	0.8
**HABITOS2. Years since starting regular smoking**			
None	A	424	85.7
Fewer than 5 years	B	21	4.2
5–10 years	C	1	0.2
11–20 years	D	8	1.6
More than 20 years	E	41	8.3
**HABITOS3. Frequency of alcoholic-beverage consumption**			
Never	A	98	19.8
Once per month or less	B	192	38.8
2–4 times per month	C	169	34.1
2–4 times per week	D	32	6.5
Four or more times per week	E	4	0.8
**HABITOS4. Number of drinks on a typical drinking day**			
1–2 drinks	A	277	56.0
3–4 drinks	B	114	23.0
5–6 drinks	C	4	0.8
7–8 drinks	D	1	0.2
10 or more drinks	E	1	0.2
Not applicable (never consumes alcohol)	0	98	19.8
**HABITOS5. Frequency of consuming six or more drinks on one occasion**			
Never	A	324	65.5
Less than once per month	B	126	25.5
Monthly	C	39	7.9
Weekly	D	4	0.8
Daily or almost daily	E	2	0.4

Questionnaire item and response category	Code	n	%
**HABITOS6. Type of habitual diet**			
Local diet	A	209	42.2
Diet from country of origin	B	112	22.6
Mixed diet	C	174	35.2
**HABITOS7. Premature cardiovascular disease in a first-degree relative**			
Yes	A	64	12.9
No	B	429	86.7
Do not know	C	2	0.4

Note: Values are presented as the number and percentage of the total study population (N = 495). Percentages may not total exactly 100% because of rounding. For HABITOS7, premature cardiovascular disease was defined as myocardial infarction, stroke, or sudden death before 55 years of age in male first-degree relatives or before 65 years of age in female first-degree relatives. For HABITOS4, participants who reported never consuming alcohol in HABITOS3 were coded as 0 (not applicable). The remaining HABITOS4 responses were retained in their original ordinal categories. Because these categories represented ranges rather than exact quantities, HABITOS4 was not analysed as a quantitative measure of alcohol intake or included as a covariate in the regression models. Current smoking was derived from HABITOS1, and alcohol-consumption frequency was derived from HABITOS3.

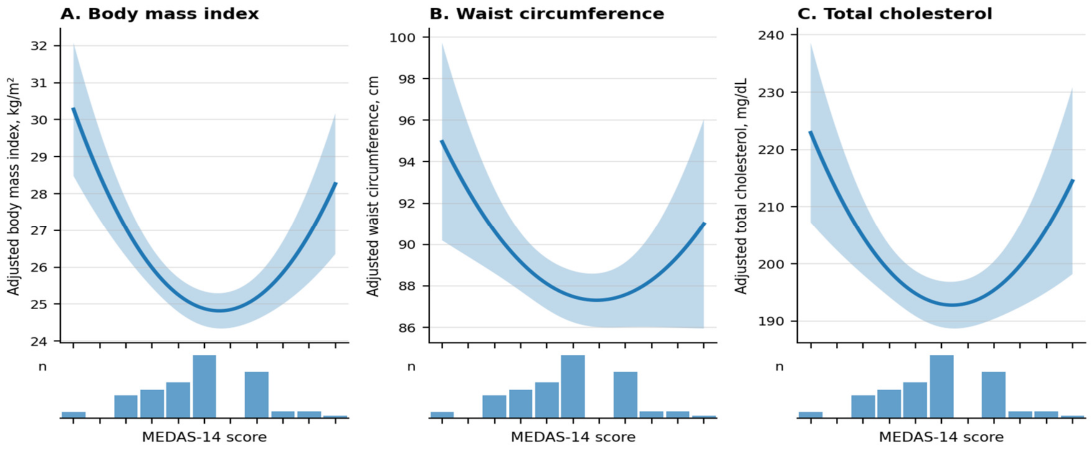

Supplementary Figure S1. Adjusted non-linear associations between the MEDAS-14 score and body mass index, waist circumference, and total cholesterol. Solid lines represent marginal adjusted predictions derived from multivariable linear regression models including a quadratic MEDAS-14 term, and shaded areas represent 95% confidence intervals. Histograms show the distribution of participants across the observed MEDAS-14 score range. Panels show (A) body mass index, (B) waist circumference, and (C) total cholesterol. Models for body mass index and waist circumference were adjusted for age, sex, educational attainment, and physical activity. The total-cholesterol model was additionally adjusted for body mass index. Quadratic terms were statistically significant for all three outcomes (body mass index, $p < 0.001$; waist circumference, $p = 0.002$; total cholesterol, $p < 0.001$). Analyses included 495 participants. MEDAS-14: 14-item Mediterranean Diet Adherence Screener.
